# Developing an assessment tool to measure health equity considerations of guideline development handbooks

**DOI:** 10.1017/S0266462326103559

**Published:** 2026-02-20

**Authors:** Ramaa Chitale, Adam Richards, Precious Williams, Tari Turner, Stephanie Goodrick, Ana González Ramos, Christina McMillan Boyles, Deana Manassaram-Baptiste, Caleb Kimutai Sagam, Eleanor Ochodo, Rachel Kowalsky, Emily Smith

**Affiliations:** 1 Department of Global Health, George Washington University, Milken Institute School of Public Health, USA; 2 School of Public Health and Preventive Medicine, Monash University, Australia; 3 National Health and Medical Research Council, Australia; 4 Spanish Scientific Research Council, Institute for Advanced Social Studies, Spain; 5 School of Nursing/École des sciences infirmières, Laurentian University, Canada; 6 American Cancer Society, USA; 7 Kenya Medical Research Institute, Kenya; 8 Centre for Evidence Based Health Care, Department of Global Health, Faculty of Medicine and Health Sciences, Stellenbosch University, South Africa; 9 NewYork-Presbyterian Weill Cornell Medical Center, USA

**Keywords:** health equity, methods, practice guideline, tool development, guideline development handbook

## Abstract

**Objectives:**

Guideline development handbooks outline the methodology that authoring organizations use to create public health and clinical practice guidelines (CPGs). We created an Equity Assessment Tool (EquAT) for guideline development handbooks to identify areas of improvement and foster conversations.

**Methods:**

Sequential phases lead to tool development and face/content validation in this mixed-methods study. In phase 1, we reviewed the literature to generate a list of “essential elements” or tasks that are part of guideline development methodology, mapped “essential elements” with relevant equity concepts, and drafted our tool for use in reviewing guideline development handbooks. In phase 2, we surveyed experts for feedback on “essential elements” and explicit language for assessing equity within the tool and refined items. We piloted and finalized the tool based on feedback.

**Results:**

We identified 18 essential elements within five domains of guideline development and created a draft EquAT. Twenty of 25 invited experts responded to the online survey for feedback on the tool. Most experts provided limited feedback, and the most common suggestion was adding clarifying language to the existing tool criteria for assessing equity. Ten experts participated in pilot testing the revised tool. We found a diversity of scores, and potential reasons might be due to the complexity of the tool, differences in equity frameworks, and a variety of expertise. We incorporated their feedback and finalized the tool.

**Conclusions:**

We developed and validated the EquAT, a tool to foster discussion among assessors about the extent of health equity considerations in guideline development handbooks.

## Introduction

Clinical practice guidelines (CPGs), as defined by the Institute of Medicine (IOM; now the National Academies), are “statements that include recommendations intended to optimize patient care that are informed by systematic review of evidence and an assessment of the benefits and harms of alternative care options” (1). Since the 1990s, CPG publication has rapidly accelerated. In 1993, PubMed introduced the Medical Subject Headings (MeSH) “practice guideline” to categorize the literature (2); there were about 800 published articles with this MeSH term in the year 2000, growing to nearly 2,000 articles by 2024. With the increase in publications, guideline-producing organizations developed methods to improve the quality and consistency of the evidence-based guideline development process (3). In 2011, the IOM published “Clinical Practice Guidelines We Can Trust,” which set standards for guideline development (1). By 2004, several authoring organizations also published guideline development handbooks to standardize the process (2). These methodological handbooks are intended to influence both the scope and quality of CPGs (2).

In addition to improving the quality and consistency of clinical care, CPGs have the potential to expand health care for all people (4), and accordingly, there have been calls to incorporate elements of health equity into guideline development handbooks. As defined by the World Health Organization (WHO), health equity is “the absence of unfair, avoidable or remedial differences among groups of people” (5). If CPGs do not at least consider differential impact by groups, guidelines may perpetuate poor health outcomes, delivery, and quality of care for groups at higher social risk (6–9). For instance, Mizen et al looked at CPGs for health problems commonly experienced by persons with intellectual disabilities. Using “The equity lens,” the researchers found that of the 36 guidelines they identified for these health problems, only eight mentioned persons with intellectual disabilities as an impacted group (7;10). Similarly, Chan et al reviewed 58 CPGs for traumatic brain injury (TBI) and two CPGs for homelessness (8). They found that few CPGs integrated sufficient evidence on the relationship between TBI and homelessness and vice versa. These systematic reviews highlight an unmet need for explicit efforts to ensure that those disproportionately affected by certain health conditions are integrated into the CPG development process.

There is growing literature on the importance of considering health equity in guideline development, and several general frameworks exist. For instance, Dans et al published “The equity lens,” to help users of CPGs, guideline developers, and policy makers consider inequities when creating, implementing, and evaluating recommendations (10). The Grading of Recommendations Assessment, Development and Evaluation (GRADE) developed a conceptual framework, including suggestions for when and how guideline developers consider health equity (11–14). These general frameworks have been essential to advancing the academic and practical work related to health equity in CPGs but do not explicitly specify when and how to consider health equity during the guideline development process.

More recently, published checklists have gone further to specify points in the guideline development process when groups may consider health equity. Lin et al present a health equity framework and checklist for the U.S. Preventive Services Task Force (USPSTF). This framework and checklist includes phase of development, specific health equity considerations, and rationale (15). In 2025, Dewidar et al published the health equity extension of the GIN-McMaster Guideline Development Checklist, to assess health equity consideration in guideline development topics, across health practice guidelines (including guideline development handbooks) (16). Although these works are informative, organizations that develop guidelines still rely on their own institutional guideline development handbook to guide panel members. Furthermore, guideline development handbooks require independent scrutiny as they represent the official stance of the authoring organization on methods.

We argue that guideline development handbooks are an essential upstream entry point for health equity considerations. Therefore, guideline authoring organizations should scrutinize whether their methods can better incorporate considerations of health equity. In this work, we developed a tool to assess health equity considerations in guideline development handbooks. To do this, we reviewed and synthesized the literature to draft the tool and then surveyed experts to ensure face and content validity. The Equity Assessment Tool (EquAT) is intended for use by guideline development authoring organizations to engage in conversation, draw on each other’s expertise, and use their institutional knowledge to identify areas within official guideline development handbooks to ensure each CPG appropriately considers health equity.

## Methods

### Intended audience

**Figure 1.**
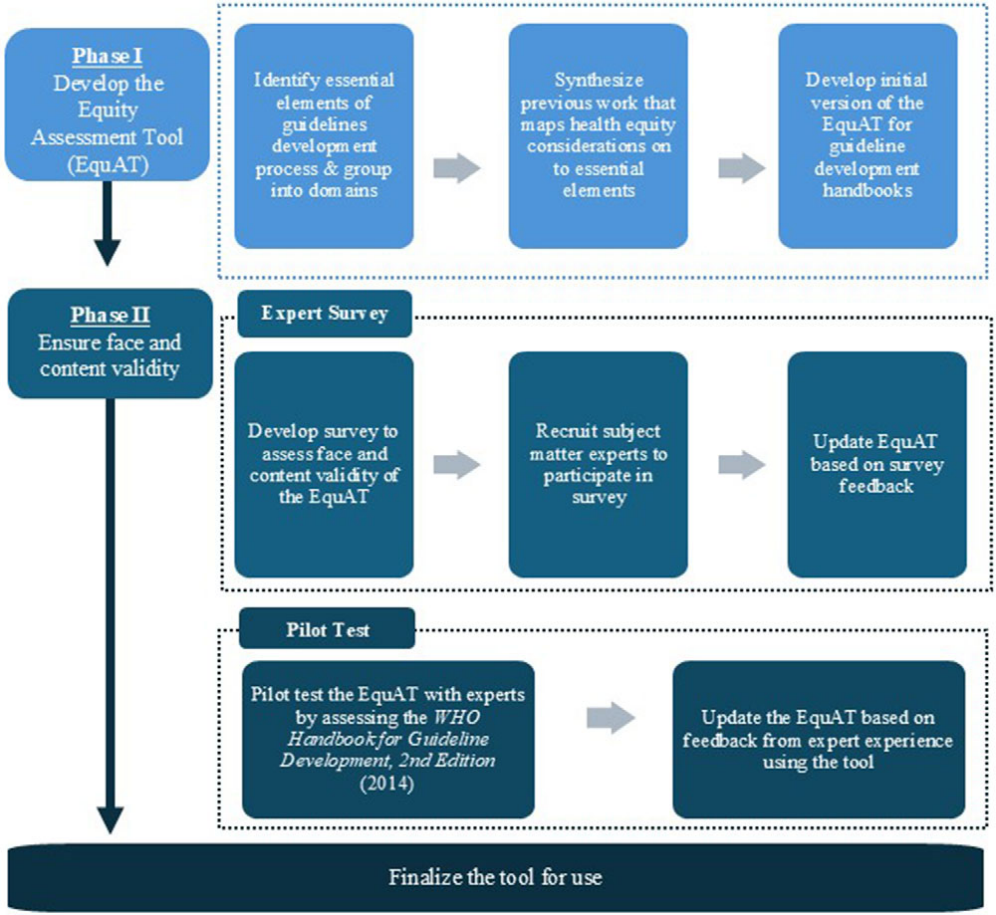
Visual representation of the two phases of development for the Equity Assessment Tool (EquAT) for Guideline Development Handbooks.

We propose an “Equity Assessment Tool” (EquAT) for guideline development handbooks. The audiences for this tool are both authors of guideline development handbooks and groups determining which work to use as part of their process. Guideline development handbook authors may use this tool to 1) audit their own guideline development handbook against practices proposed in the tool and 2) foster discussions among themselves on ways to enhance health equity considerations in their guideline development processes. We developed and conducted face/content validity of the EquAT tool in sequential phases. Figure 1 is a visual representation of our process.

### Phase I – Review and synthesis of literature to develop an equity assessment tool

#### Identification of the essential elements of guideline development handbooks

We reviewed published literature to identify essential elements of guideline development methodology based on published literature. We defined “essential elements” as the tasks that scholars generally agree should be part of all guideline development (2). We identified three main assessment frameworks related to guideline development handbooks. The Guideline International Network and McMaster University (GIN-McMaster) guideline development checklist includes 18 topics of guideline creation (17). We triangulated this checklist with two additional publications that focus solely on guideline development handbooks (2;17;18). Turner et al identified 14 key elements from the literature and 6 guideline development handbooks. Ansari et al used 19 guideline development handbooks to identify 27 main tasks. Tasks from all three publications are in Supplementary Material (S1). We compared these and grouped similar tasks to develop a comprehensive list.

#### Synthesis of evidence to create a tool

One researcher (RC) synthesized literature that mapped these tasks, now described as “essential elements” of guideline development, with health equity considerations drawn from a 2023 scoping review of published best practices for equity inclusion in guideline development (19), along with three publications from the GRADE group, and commonly referenced guideline development handbooks (11,12,14). Criteria for assessing equity were drawn from Shaver et al, which summarizes the “best practices for considering health equity in each stage of the guideline development process” (19). The three GRADE publications provide specific suggestions about how and when to consider health equity during the guideline development process. We chose these publications since groups commonly use GRADE methods in evaluating the quality of evidence and strength of recommendations (20). During our search for relevant literature, we also identified experts based on authorship of peer-reviewed publications relevant to equity considerations in guideline development. We spoke with these individuals about strategies to improve health equity considerations during the guideline development process. The research team grouped essential elements into domains. The literature review and conversations with experts informed the further development of the tool.

#### Tool development

We based the structure of the EquAT tool on the Appraisal of Guidelines for Research and Evaluation, second edition tool (AGREE II) (21). The original AGREE tool was developed in 2003 to assess the “process of guideline development and reporting of this process in the guideline” (21). Our tool initially included the following fields: the name of the essential element, definition of essential element, a 5-point assessment scale (1 = meets none of the criteria nor considerations, 5 = meets all criteria and considerations), criteria, considerations, where to find the essential element within a guideline development handbook, exemplar quotations from other guideline development handbooks that are rated highly, and relevant abbreviations. Criteria refer to explicit equity-focused language or subject matter that an assessor should look for when reviewing a guideline development handbook. Considerations supplement these criteria and may be used to help the assessor decide on a score. Considerations ask the assessor if 1) the equity criteria are easy to find within a given guideline development handbook and 2) if the equity criteria themselves are clearly worded.

### Phase II – Ensuring face and content validity of the equity assessment tool for guideline development handbooks

To ensure face and content validity, we first conducted a survey to elicit expert feedback on the tool. The intent of the survey was to confirm that our tool was indeed assessing health equity, according to experts (face validity). Further, the surveys helped identify how well the items in the tool capture the breadth of health equity considerations in guideline development (content validity). Next, we piloted the application of the tool by assessing the *World Health Organization handbook for guideline development, 2nd Edition* (2014) with a group of experts (22). The pilot test helped identify areas of further refinement.

#### Survey participant selection

We identified experts who had either 1) previously contributed to a guideline development handbook, 2) participated in a guideline development panel, 3) published peer-reviewed research articles on health equity and guideline development, or 4) were current students or post-docs researching this topic. Over email, we asked these experts to participate in our survey and provide feedback for our tool. The George Washington University Institutional Review Board (IRB) (NCR235308) determined this survey to be exempt from ethical review.

#### Survey design

We developed five domain-specific online surveys (Supplementary Material (S2)) using the *SurveyMonkey* platform to elicit feedback open from May to August 2024. We allocated participants to respond to one of the five surveys based on the topic of their previous publications. For example, if a participant had previously researched guideline panel make-up, we allocated them to the survey for domain 2 “Identifying the guideline group.” For each essential element, we asked experts to rate how relevant the listed criteria were in assessing a guideline development handbook for equity inclusion on a 9-point Likert scale (1 = clearly not relevant, 9 = clearly relevant). We also asked two open-ended questions for each essential element: “Do you have feedback on additional criteria?” to those we listed and “Do you have suggestions to improve the clarity of these criteria?” After reviewing feedback, we generated thematic codes to categorize responses and incorporated suggestions into the tool.

In the same surveys, we showed experts quotations from frequently cited guideline development handbooks to demonstrate examples of language and content that we thought explicitly mentioned equity for each “essential element.” In the final version, these quotes would serve as positive examples of health equity-focused strategies. We chose example quotes based on the survey design team’s perception of highly equity-focused quotations. We asked experts to rate how equity-focused a given quote was on a 5-point Likert scale (1 = not at all equity focused, 5 = highly equity focused). We asked the open-ended question, “Do you think this is a good example of a highly equity-focused quotation?” and “Do you have another example in mind that might be better?” When reviewing these quotations, we did not ask experts to score their responses based on the criteria that match the “essential element” from the EquAT tool. If experts disagreed with any of the criteria, we did not want to influence their scoring of the quotations. We originally planned a discussion with experts to settle disagreements identified in the survey, as commonly done in the Delphi process. However, after analyzing the surveys, and noting a few disagreements, we decided to pilot test the tool instead. We modified the tool based on survey feedback.

#### Pilot testing

Next, we invited the same group of experts to pilot test the EquAT tool by assessing the *World Health Organization Handbook for Guideline Development, 2nd Edition* (2014) (22). We chose this work since the WHO creates broad public health and CPGs applied worldwide. Therefore, we expected that the WHO would account for equity in their guideline development handbook.

We provided each expert with 1) the updated EquAT tool, 2) the *WHO Handbook for Guideline Development*, including supplemental chapters, and 3) instructions (Supplementary Material (S3)). As the *WHO Handbook for Guideline Development* is lengthy, the instructions included a chart on relevant sections, to save expert time. We asked the experts to apply the EquAT and provide any feedback in a comments section on how the tool might be improved. We reviewed their responses, considered and resolved conflicting responses, and incorporated additional feedback into the final tool. Additionally, to further understand tool performance, we spoke with the experts who participated in the pilot test about their experiences in applying the tool.

## Results

### Identification of essential elements and drafting the tool

Our review and synthesis of the literature identified 18 tasks, “essential elements,” of guideline development (Table 1), and we categorized them into five domains: preparing for the guideline, identifying the guideline group, gathering the evidence, drafting and review, and dissemination, implementation, evaluation, and updating. We mapped these essential elements with health equity considerations from previous publications and drafted the first version of the tool.

**Table 1.** List of 18 essential elements under five domains of guideline development identified by the author team by comparing three previous publications

Domain	Essential element number and title^a^
Domain 1: Preparing for the guideline	#1 Defining a topic
	#2 Determining the scope
	#3 Identifying existing guidelines, adapting, and adopting
Domain 2: Identifying the guideline group	#4 Forming guideline development group
	#5 Involving special subgroups
	#6 Managing conflicts of interest
Domain 3: Gathering the evidence	#7 Establishing clinical questions
	#8 Outcome consideration
	#9 Systematic search for evidence and evidence inclusion
	#10 Summarizing the evidence
	#11 Appraising the evidence
	#12 Conducting economic evaluation
Domain 4: Drafting and review	#13 Creating recommendations
	#14 Wording and considering ethics
	#15 Reporting and consulting experts
Domain 5: Dissemination, implementation, evaluation, and updating	#16 Dissemination and publications
	#17 Implementation and evaluation
	#18 Updating

a Essential elements are tasks that scholars generally agree are an integral part of the guideline development methodology.

### Survey respondents

We surveyed experts and asked them to review our draft tool to 1) interpret the relevance of the health equity criteria for each essential element and 2) rate sample quotations we had selected to demonstrate what a guideline with exemplary language might look like. We invited 25 experts to participate in the survey, and 20 completed the online survey. Their locations included the United States (7), Australia (5), Canada (3), Kenya (2), Denmark (1), South Africa (1), and Spain (1). Twelve respondents described themselves as guideline development methodologists, eight respondents as guideline development handbook contributors, eight as health equity researchers with publications about guideline development, six as guideline development panel members in the past or present, two experts described themselves as systematic reviewers for guideline development groups, and one reviewer was a current student or postdoctoral researcher conducting work on health equity and guideline development.

### Survey results – feedback on the tool

Three to six experts reviewed each of the domains; six experts reviewed domain 1, four experts reviewed domain 2, four experts reviewed domain 3, three experts reviewed domain 4, and three experts reviewed domain 5. In general, we found that experts scored the equity criteria for each essential element as highly relevant (Table 2). Scores for equity criteria for “essential elements” ranged from 3 to 9 (scale: 0 = clearly not relevant, 9 = clearly relevant). Out of all the scores for all essential elements (72 possible scores), 86 percent of scores were greater than the midpoint of five.

**Table 2.** Summary from online survey asking experts to rate equity criteria of the tool or explicit equity-focused language to look for within a guideline development handbook

Domain number	Essential element number and title^a^	Number of reviewers	Range of scores^b^	Mean	Domain range	Domain mean
1	#1 Defining a topic	6	4–9	7	4–9	7
	#2 Determining the scope	6	4–9	7		
	#3 Identifying existing guidelines, adapting, and adopting	4	4–9	8		
2	#4 Forming guideline development group	4	7–9	9	6–9	8
	#5 Involving special subgroups	4	7–9	8		
	#6 Managing conflicts of interest	3	6–9	8		
3	#7 Establishing clinical questions	4	8–9	8	3–9	8
	#8 Outcome consideration	4	5–9	8		
	#9 Systematic search for evidence	4	7–9	8		
	#10 Summarizing the evidence	4	4–9	8		
	#11 Appraising the evidence	4	6–9	8		
	#12 Conducting economic evaluation	4	3–8	7		
4	#13 Creating recommendations	3	7–8	8	4–9	7
	#14 Wording and considering ethics	3	5–9	7		
	#15 Reporting and consulting experts	3	4–7	6		
5	#16 Dissemination and publications	3	5–8	6	3–8	6
	#17 Implementation and evaluations	3	3–6	6		
	#18 Updating	3	6–7	7		

a Essential elements are tasks that scholars generally agree are part of all guideline development.

b Survey question: “Consider these criteria all together. If you were to assess a guideline development handbook for equity inclusion, how relevant are the listed criteria?” Scale of assessment is 1–9 where 1 = Clearly not relevant, 9 = Clearly relevant.

Definition of thematic codes and examples of feedback for open-ended questions in the survey are in Supplementary Material (S4). We found that the most common feedback was “no feedback.” The second most common code applied to the data was a suggestion from the respondents to provide “additional language to existing criteria.” There were instances where we were unclear about how to change the tool based on expert feedback. For example, one expert noted that the “criteria are quite high level and don’t provide the practical guidance…” (domain 2, essential element #4). In these instances, we tried to resolve the comments by triangulating other participants’ feedback and updating the tool accordingly. Additionally, we took one expert’s suggestion to change the assessment scale from 1–5 to 0–4, to intuitively account for the fact that guideline development handbooks that do not have any equity considerations receive no points.

### Survey results – feedback on exemplary quotations

**Figure 2.**
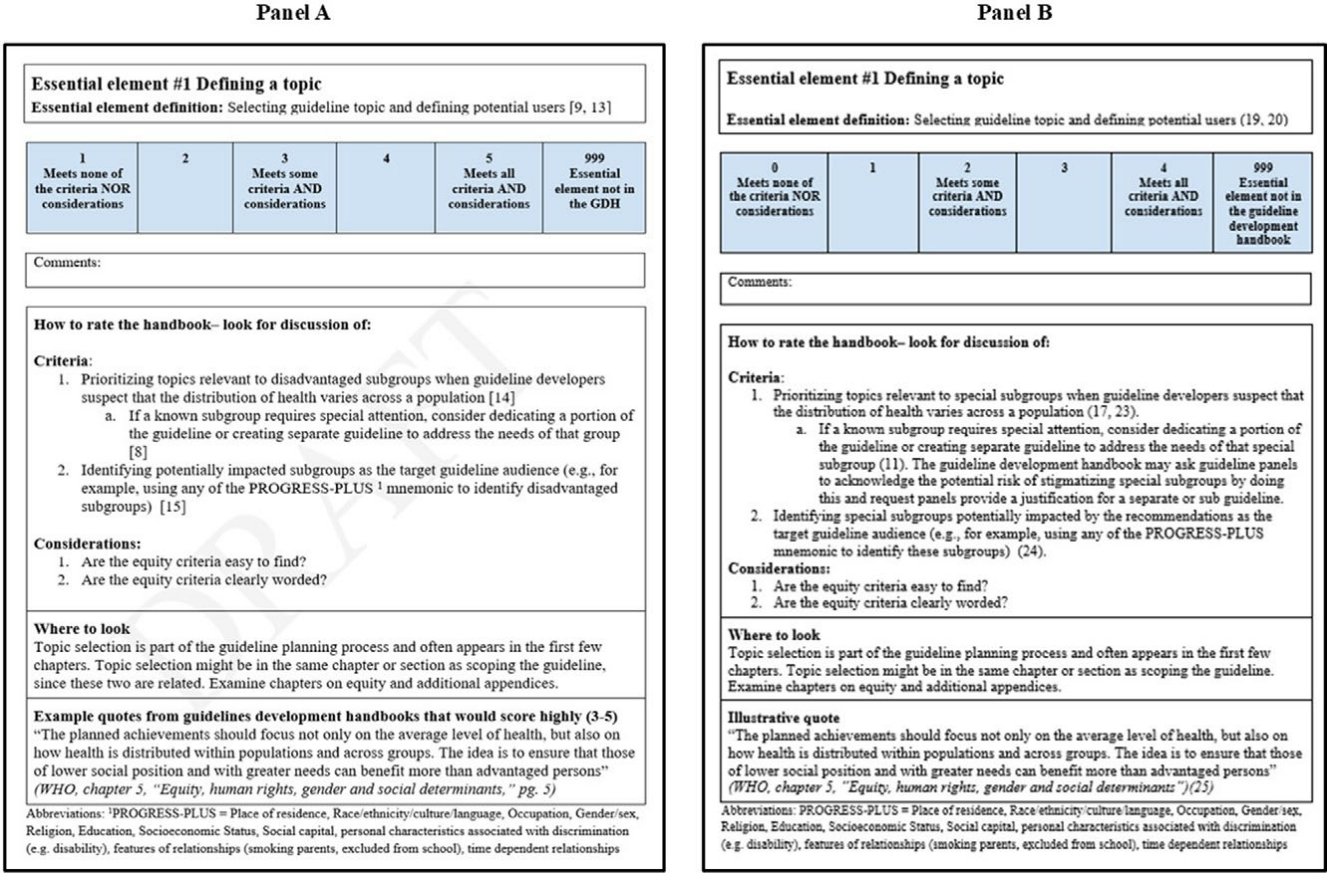
Difference between the first and third versions of tool development after expert input through survey and pilot testing. Panel A is the first version, and panel B is the final version.

We present expert ratings of what we initially termed “highly rated quotations” in Supplementary Material (S5). We found no clear patterns in expert feedback. In Supplementary Material (S6), we show example quotations, scores, and interpretations from the experts who reviewed essential element #4; two experts suggested ways that the guideline development handbook improves their language, whereas the other two experts provided little or no feedback. Other survey responses followed a similar pattern, with a diversity of opinions on whether the quotations were good examples of equity-focused language. We modified the EquAT accordingly. Previously, we called this section in the tool “quotations from handbooks that are rated highly.” However, we changed the title of this section in the tool to “illustrative quotes” to ensure we did not misrepresent any views on the language (Figure 2B). We created a revised tool based on the survey and other feedback for pilot testing (Figure 2B).

### Pilot test results

Ten experts participated in the pilot application of the tool to the *WHO Handbook for Guideline Development.* The goal of the pilot was to gather feedback after implementation of the tool and note any points of confusion.

When using the tool, experts assessed some essential elements in the *WHO Handbook for Guideline Development* similarly, whereas other essential elements had disagreement by one or more experts. We defined disagreement as ratings that deviated by 2 or more points from the mean. The heat map (Table 3) shows scores from each expert, mean, and range for each essential element. Scores above the mean are indicated in green and below the mean are indicated in orange. We found that expert #5 gave 12 ratings that differed from the mean by 2 or more points. Excluding expert #5, other essential elements that had disagreement were #6 Managing conflicts of interest (expert #2), #8 Outcomes consideration (expert #7), #10 Summarizing the evidence (expert #1), #14 Wording and considering ethics (expert # 9), #15 Reporting and consulting experts (expert #4), #16 Dissemination and publications (expert #7), #17 Implementation and evaluation (experts # 4 and # 9), and #18 Updating (expert #7). Expert #8 assessed only the first six essential elements, and we included them for these essential elements.

**Table 3.** Scores from 10 experts for each essential element during the pilot test where they applied the EquAT tool to the WHO Handbook for Guideline Development, 2^nd^ Edition

Domain number	Essential element number and title	Range^a^	Mean^b^	Expert 1^c^	Expert 2^c^	Expert 3^c^	Expert 4^c^	Expert 5^c^	Expert 6^c^	Expert 7^c^	Expert 8^c^	Expert 9^d^	Expert 10^d^
1	#1 Defining a topic	3–4	3	3	4	3	3	3	3	3.5	4	3	3
	#2 Determining the scope	1–4	3	4	4	3	4	**1**	3	2	4	2	3
	#3 Identifying existing guidelines, adapting, and adopting	0–4	3	2	3	3	4	**0**	2	–	3	2	2
2	#4 Forming guideline development groups	0–4	3	3	4	3	4	**0**	3	2.5	4	3	3
	#5 Involving special subgroups	2–4	3	3	2	3	4	**2**	3	2.5	3	2	4
	#6 Managing conflicts of interest	0–4	3	3	**1**	4	2	**0**	4	2	4	2	4
3	#7 Establishing clinical questions	2–4	4	3	4	4	4	**2**	4	4	–	4	4
	#8 Outcome consideration	1–4	3	3	3	4	4	2	3	**1**	–	3	4
	#9 Systematic search for evidence	0–4	4	3	4	4	4	**0**	4	4	–	3	4
	#10 Summarizing the evidence	1–4	3	**1**	3	4	4	2	3	2	–	2	3
	#11 Appraising the evidence	0–4	4	3	4	4	4	**0**	4	3	–	4	4
	#12 Conducting economic evaluation	–	–	–	–	–	–	–	–	–	–	–	–
4	#13 Creating recommendations	2–4	4	3	4	4	4	**2**	3	3	–	3	4
	#14 Wording and considering ethics	1–4	3	3	3	4	3	4	3	3	–	**1**	3
	#15 Reporting and consulting experts	0–4	2	1	2	3	**4**	**0**	2	1	–	1	2
5	#16 Dissemination and publications	0–4	3	3	4	4	4	**1**	3	**0**	–	3	4
	#17 Implementation and evaluations	0–4	2	2	2	2	**4**	**0**	2	2	–	**0**	2
	#18 Updating	0–3	2	1	2	2	3	2	2	**0**	–	2	3

*Note:* Blue cells indicate experts gave an average score, green cells indicate higher than average, and orange cells indicate lower than average. Cells that are darkened mean disagreement (defined as 2 or more points away from the mean). Cells in white indicate the essential element was not scored.

a Each essential element for the guideline development handbook was scored on a 5-point scale (0 = Meets none of the criteria or considerations, 2 = Meets some of the criteria and considerations, 4 = Meets all criteria and considerations).

b Mean (rounded to 0 significant figures) is calculated without expert #5, as they consistently gave essential elements lower scores.

c Experts #1–8 are individuals who have previously contributed to a chapter or the entire guideline development handbook, participated in a guideline development panel, or published peer-reviewed articles on health equity and guideline development.

d Experts #9–10 are current students researching this topic.

To understand this diversity in pilot reviewer assessments, we reviewed their comments. Experts did not always have uniform interpretations of language in the *WHO Handbook for Guideline Development.* For instance, expert #1 was categorized as having disagreement with the other reviewers for essential element #10, “Summarizing the evidence”; they said, “Considering how stringent GRADE is, more information on equity consideration is needed in evidence synthesis,” whereas others felt satisfied with the language of the guideline development handbook for this essential element seen by the tighter range of scores (2–4) in Table 3.

To further understand the reasoning for disagreements, we had one-on-one discussions with experts (#1, #2, #7, #8). Individuals noted that complex thinking and overly detailed instructions may have contributed to a diversity of interpretations in the guideline development handbook. Experts had difficulty translating written criteria from the tool to the relevant sections of the guideline development handbook. They also noted that their current work, individual backgrounds, and biases might have influenced how critically they looked for health equity consideration in a particular section of the guideline development handbook.

To address observations from these experts, we added a section in the instructions of the EquAT tool guiding questions. Before assessors apply the tool, they should consider 1) the meanings of health equity in their organization, 2) the experience and areas of emphasis for the assessment team, and 3) identify essential elements that the group does not want to assess. By adding these discussion points, we hope that groups emphasize transparency in their assessment process. We present our finalized tool in Supplementary Material (S7).

## Discussion

We created the EquAT tool to assess the extent to which health equity considerations are addressed in guideline development handbooks. The purpose of this tool is not to generate a score nor to compare one handbook to another. Rather, this tool is to provide developers/users of guideline development handbooks a way to easily identify opportunities for improvement and enable informed conversations about possible ways to address health equity in their guideline development. As guideline development handbooks are one upstream entry point for health equity considerations, we hope that conversations enabled by our tool help authoring groups update and refine their guideline development handbooks. In turn, perhaps the processes for developing future public health and CPGs should better consider health equity.

Generally, experts agreed on how to incorporate health equity considerations into the stages of guideline development. However, we found some variation in scoring when applying our tool. Potential reasons include preconceived expectations of equity-focused language, and expertise in particular areas may have led to stringent scoring for those areas. Given these variations and recognition that the guideline development process is complex, we appreciate that having precise criteria does not always make sense. As such, in our assessment instructions, we do not specify the number of criteria that a guideline development handbook must meet to obtain a certain score. This furthers the goal of fostering conversations about which essential elements and corresponding equity criteria are most relevant to the organization.

We recognize that expert opinion during the guideline development process remains powerful despite an emphasis on evidence-based guideline development. Those who evaluate the guideline development handbook might have institutional knowledge about their authoring organizations and the context of guideline application. They may bring unique views to their assessment based on their individual emphasis. These qualities add value to the tool as experts can discuss their assessments together, compare their interpretations of health equity, and use this information to improve their guideline development handbook. In our study, only 20 experts provided feedback. Of those 20, only 3–6 experts provided feedback for each domain. To improve reliability, future research might include additional input from experts into the criteria of each essential element, as well as qualitative analysis of group discussions. The benefit of more voices is apparent in Table 3; for instance, essential element #1 had the tightest range of scores during the pilot test and had the greatest number of experts (6) during the survey portion of tool development.

Given our findings, we updated our instructions to suggest that reviewers discuss the meaning of health equity and their expertise to create a shared goal of the assessment prior to using the EquAT. For instance, in our pilot, two experts lived in the same country and referenced the same guideline development handbook for their research. However, they disagreed by more than 2 points from the mean on four of the essential elements. These two experts research in areas that emphasize different parts of guideline development. A discussion between these experts before and after the assessment may target areas of the guideline development handbook that need enhancement.

## Limitations

Our study had limitations in both the tool development process and the expert review process. First, we referenced publications on guideline development in high-income country contexts to create our tool. Common handbooks come from high-income country contexts like North America, Australia, and Europe. As a result, our tool may be more relevant for high-income country guideline development handbooks. In low- and middle-income countries, guidelines are sometimes adapted, adopted, or contextualized based on high-income country guidelines, partially because of insufficient resources, access to evidence, and training to develop new guidelines (23;24). Future research might modify the tool for guideline development handbooks that are about adapting/adopting/contextualizing guidelines.

The drivers of health equity (like socioeconomic status or race) may differ based on location, a concept several experts highlighted during the pilot test. This difference in context and common examples of inequity might impact how experts determine “which measurements are monitored by national, state/provincial, and local governments and international agencies” and which activities receive resources (25). Our tool would be less helpful for guideline development authoring organizations if there is no shared meaning of health equity among those who create guideline development handbooks (26). To address this limitation, we added guiding questions to the instructions portion of the EquAT.

Due to the length of the tool, we chose a 5-point Likert scale for rating equity criteria. Users must discern a difference between assessment numbers, which threatens the reliability of the tool and may lower performance (21). For example, one expert scored using decimal points to capture nuances during piloting. Also, though it would have been beneficial, we did not ask any expert to provide feedback on the entire tool during the survey round to improve feasibility for participation. Instead, we tried to match expertise with the most relevant domain in the tool.

## Conclusion

The guideline development process is rigorous, complex, location, and organization-dependent. Developers must consider diverse viewpoints, experiences, and contexts. Future work includes applying EquAT to other national and international guideline development handbooks to identify opportunities for equity enhancement through guided discussion with assessors.

## Supporting information

10.1017/S0266462326103559.sm001 Chitale et al. supplementary material Chitale et al. supplementary material

## Supplementary material

The supplementary material for this article can be found at http://doi.org/10.1017/S0266462326103559.
