# Supplementary material for: Developing an assessment tool to measure health equity considerations of guideline development handbooks
Source: Int J Technol Assess Health Care. 2026 Feb 20;42(1):e28. doi: 10.1017/S0266462326103559 (PMC13071845; doi:10.1017/S0266462326103559)
Supplement: Chitale et al. supplementary material [file S0266462326103559sup001.pdf]

Developing an assessment tool to measure health equity considerations of guideline development handbooks

Ramaa Chitale, Adam Richards, Precious Williams, Tari Turner, Stephanie Goodrick, Christina McMillan Boyles, Ana González Ramos, Deana Manassaram-Baptiste, Caleb Kimutai Sagam, Eleanor Ochodo, Rachel Kowalsky, Emily R. Smith

Supplementary file

Correspondence to: Ramaa Chitale DrPH, ramaa.chitale@gmail.com

Supplementary files

S1 –Three previous publications on tasks involved in guideline development
S2 –Five domain specific surveys for expert feedback on equity assessment tool
S3 - Instructions for pilot testing the EquAT
S4 - Thematic codes applied to feedback from survey data open ended responses
S5 - Ratings of experts on equity focused quotations from guideline development handbooks
S6- Reactions of four reviewers to an example of an equity focused quotation from a guideline development handbook
S7- Final EquAT tool

S1 – Three previous publications on tasks involved in guideline development

S1- Three previous publications on tasks involved in guideline development

Turner et al (2008)	Ansari et al (2012)	Schünemann et al 2014
• Selecting the topic	• Selecting the guideline topic	• Organization, budget, planning and training
• Determining the scope of the CPG	• Determining the guideline scope	• Priority setting
• Identifying and adapting the existing CPG	• Preparing the work plan	• Guideline group membership
• Forming a multidisciplinary guideline development group	• Identifying relevant existing guidelines	• Establishing guideline group process
• Involving consumers	• Appraising relevant existing guidelines	• Identifying target audience and topic selection
• Establishing clinical questions	• Adapting existing guidelines	• Consumer and stakeholder involvement
• Systematic searching (including documentation of sources, filters, and limits)	• Involving consumers	• Conflict of interest consideration
• Including and/or excluding identified research	• Forming guidelines development group	• Question generation
• Appraising research	• Managing conflict of interest	• Considering importance of outcomes and interventions, values, preferences and utilities
• Developing recommendations	• Running guideline development group	• Deciding what evidence to include and searching for evidence
• Developing an implementation strategy	• Developing clinical questions	• Summarizing evidence and considering additional information
• Consulting on the draft CPG	• Systematic search for evidence	• Judging the quality, strength or certainty of a body of evidence
• Writing of summary versions of the CPG	• Selecting relevant evidence	• Developing recommendations and determining their strength
• Planning for evaluating the impact, revising and updating the CPG	• Appraising identified research evidence	• Wording of recommendations and of considerations about implementation, feasibility, and equity
	• Evidence synthesis and analysis	• Reporting and peer review
	• Conducting economic evaluation	• Dissemination and implementation
	• Making group decisions	• Evaluation and use
	• Grading available evidence	• Updating
	• Considering ethical issues	
	• Creating recommendations	
	• Final stakeholder consultation	
	• Publishing formats	
	• Guideline implementation strategies	
	• Piloting	
	• Assessment the potential impacts of guideline implementation	
	• Developing clinical audit and evaluation criteria	
	• Updating recommendations and correcting potential errors	

S2 – Five domain specific surveys for expert feedback on equity assessment tool

Informed consent

We (Ramaa Chitale, doctoral student, MPH, and Emily Smith, Associate Professor, ScD) are inviting you to take this survey for research. Participation is completely voluntary and there are no negative consequences if you do not participate in this survey. If you start the survey, you can stop at any time without completing it. At the end of this survey, we may ask you to participate in a second round of expert feedback, which involves an online Zoom group discussion. This is also voluntary. You are free to accept or decline participation in this follow up online Zoom discussion.

What is the purpose of this research?
The purpose of this study is to elicit expert feedback on a new tool that assesses equity considerations in guideline development handbooks for public health/clinical practice guidelines.

What will I do?
There are two types of questions for this survey. First read items from a new tool developed for the purpose of this study. Second, read a quotation from a commonly referenced guideline development handbook. There are 3 to 4 questions about each item in the tool. The survey will take about 45 min – 1 hour.

After this survey, you may be asked to participate in a second round to further elicit expert feedback on the new tool. The second round will be an online Zoom group discussion, which will last 1-2 hours.

Risks

- You might disagree with the way your organization handles equity in guideline development. However, none of these questions ask you about your role in guideline development. Instead, all questions are about your own opinions.
- Online data being hacked or intercepted: any time you share information online there are risks. We are using Survey Monkey, an online survey platform and George Washington University partnered Zoom account, and George Washington University Box account, but we cannot eliminate this risk entirely.
- Breach of confidentiality: there is a chance that your data could be seen by someone who is not meant to. We are minimizing the risk in the following ways:

- We will store all electronic data on a password protected encrypted computer.
- We will keep your identifying information separate from the research data, but we will link it to you. We will destroy this link after we are finished collecting and analyzing the data.
- If you participate in the second round of the survey, we will destroy the recording after collecting and analyzing the data from the online discussion.

Possible benefits: There is no direct benefit for you. However, your contributions will help to create a tool that may help organizations assess their own documents and understand the extent to which they do/do not consider health equity. In the future, these organizations may update their guideline development handbooks based on the discussions about the assessment tool.

Estimated number of participants: For the first round of the study, we will invite up to 40 participants for the online survey. We will then invite 20 participants of these original 40 back to the online Zoom group discussion.

How long will it take? This survey should take you no more than 1 hour. If you participate in the second round of expert feedback, this online discussion should take no more than 2 hours.

Costs: It will not cost you anything to participate in this study.

Compensation: You will not receive any compensation for participating in this study.

Future research: Your data will not be used or shared with any researchers or used for other future studies.

Confidentiality and data security: We collect the following information: name, email address, and job category. We also ask if you would like to be acknowledged in this work. This information is necessary to characterize participants.

Where will the data be stored: Data from the survey will be downloaded from SurveyMonkey (an online survey platform) and stored on the researcher's George Washington University Box account. For the second round of expert feedback (group discussion), researchers will use a George Washington University Zoom account to record the meeting, and store this recording on the George Washington University Zoom platform.

How long will it be kept? The Zoom recording will be destroyed after 6 months. The survey data and meeting notes will be kept 6 years after analysis.

Who can see my data?

- We (the researchers) will have access to identifiable (with your name included) data. This is so that we can analyze the data and conduct the study.
- We may share our findings in publications or presentations. If we do, results will be aggregated, with no identifiable information. If we quote you, we will use pseudonyms (fake names).

Questions about research, complaints, or problems: Contact Ramaa Chitale, rchitale@gwmail.gwu.edu 202-413-1712 or Emily Smith emilysmith@gwmail.gwu.edu 202-994-3589

Questions about your rights as a research participant, complaints, or problems: Contact George Washington University IRB (Institutional Review Board) at 202-994-2715 email: ohriirb@gwu.edu

Agreement to participate

Your participation is completely voluntary, and you can withdraw at any time. To take the survey you must be:

- At least 18 years old

If you meet the criteria and would like to take the survey, click the button below to start.

Equity in guideline development handbook - Domain 1

General information

* 1. Name

2. Current employer or academic institution

3. Which of the following describes your experience (select all that apply)?

- [ ] Guideline development panel member in the past or present
- [ ] Guideline development methodologist
- [ ] Health equity researcher with publication(s) about guidelines
- [ ] Guideline development handbook contributor (either helped develop a chapter or the entire guideline development handbook)
- [ ] Current student or postdoc conducting work either on health equity or guideline development
- [ ] Other (please specify)

* 4. Are you willing to be acknowledged in this dissertation and the accompanying peer reviewed manuscript?

- ( ) Yes
- ( ) No
- ( ) Not sure, please contact me to discuss further

Equity in guideline development handbook - Domain 1

Instructions for assessment

In the next **3** questions, I will show you a portion of the tool on Guideline Planning (defining a topic, determining the scope, identifying existing guidelines, adapting, and adopting). If you would like to see the entire draft tool, please see the document attached to the email.

1) Read the "essential element" name, definition, and how to rate the guideline development handbook text.

a. The term "essential element" refers to methodological components that scholars generally agree are part of the guideline development process. <u>You will only see 3 "essential elements," but a full list of all essential elements is available in the draft tool.</u>

2) Rate how equity focused you think the criteria are and provide additional feedback. Consider the criteria together. Criteria detail explicit language or subject matter that an assessor should seek when assessing the guideline development handbook.

****Remember, this tool is not for guidelines themselves, but for the guideline development handbooks that describe guideline development methodology**.

Equity in guideline development handbook - Domain 1

Essential element #1

Essential element #1 – Defining a topic

Essential element definition: Selecting guideline topic and defining potential users

How to rate the handbook – look for discussion of:

Criteria:

1. Prioritizing topics relevant to disadvantaged subgroups when guideline developers suspect that the distribution of health varies across a population.
   a. If a known subgroup requires special attention, consider dedicating a portion of the guideline or creating separate guideline to address the needs of that group.
2. Identifying potentially impacted subgroups as the target guideline audience (e.g., for example, using any of the PROGRESS-PLUS[1] mnemonic to identify disadvantaged subgroups).

Abbreviation: PROGRESS PLUS= Place of residence, Race/ethnicity/culture/language, Occupation, Gender/sex, Religion, Education, Socioeconomic Status, Social capital, personal characteristics associated with discrimination (e.g. disability), features of relationships (smoking parents, excluded from school), time dependent relationships

5. Consider these criteria all together. If you were to assess a guideline development handbook for equity inclusion, how relevant are the listed criteria?

1 = Clearly not relevant	2	3	4	5	6	7	8	9 = Clearly relevant
○	○	○	○	○	○	○	○	○

6. Do you have feedback on additional criteria?

7. Do you have suggestions to improve the clarity of these criteria?

Equity in guideline development handbook - Domain 1

Essential element #2

Essential element #2 – Determining the scope

Essential element definition: Developers search literature to create a framework that describes the epidemiology of the disease or condition and "aspects of care and the setting [that] is covered by the guideline" (Ansari et al, 2012)

How to rate the handbook– look for discussion of:

Criteria

1. A scoping literature review which includes:
    a. Determining the importance or non-importance of equity to guideline development.
    b. If the distribution of health effects varies within a population, identifying impacted subgroups as the target audience.
    c. Investigating harmful impacts of an action/intervention on population subgroups.
2. Requiring input on guideline scope from 1) members of affected subgroups or 2) other experts.
3. Reviewing literature with a lens of distribution of health (health inequities), not just average health effects, to identify the scope of the topic.

8. Consider these criteria all together. If you were to assess a guideline development handbook for equity inclusion, how relevant are the listed criteria?

1 = Clearly not relevant	2	3	4	5	6	7	8	9 = Clearly relevant
○	○	○	○	○	○	○	○	○

9. Do you have feedback on additional criteria?

10. Do you have suggestions to improve the clarity of these criteria?

Equity in guideline development handbook - Domain 1

Essential element #3

Essential element #3 – Identifying existing guidelines, adapting, and adopting

Essential element definition: Developers search for previous guidelines on the same topic in relevant databases to ensure they are not duplicating work. Guideline developers describe adaptation/adoption methods for other populations and contexts.

How to rate the handbook– look for discussion of:

Criteria

1. Searching for previously published guidelines on the same topic to avoid duplication.
2. Considering if previously published guidelines accounted for equity factors (e.g., if previous guideline content included distribution of health, variation in health services, and outcomes, and identifying target subgroups).
3. When adapting/adopting a guideline
   a. Considering local context, needs, and differences with the original guidelines
   b. Selecting an appropriate guideline panel and/or advocating for co-creation of guideline
   c. Offering methodological training for guideline developers during adaptation/adoption phase

11. Consider these criteria all together. If you were to assess a guideline development handbook for equity inclusion, how relevant are the listed criteria?

1 = Clearly not relevant	2	3	4	5	6	7	8	9 = Clearly relevant
○	○	○	○	○	○	○	○	○

12. Do you have feedback on additional criteria?

13. Do you have suggestions to improve the clarity of these criteria?

Instructions for quotation questions

Examples of equity focused quotations from other guideline development handbooks may help organizations model their own language.

In the next **6** questions, I will show you a quotation from a guideline development handbook. These quotes are from the guideline planning portion (defining a topic, determining the scope, identifying existing guidelines, adapting, and adopting) of several commonly referenced guideline development handbooks.

Read each quotation and provide feedback.

Please note that some of these quotations are long because I did not want to take them out of context. However, I recognize that this is not fully possible, and is a limitation of the survey format.

The goal of these next questions is to identify equity-focused language that would help a future tool user know what to generally look for.

Quotation 1 - Defining a topic

“The planned achievements should focus not only on the average level of health, but also how health is distributed within populations and across groups. The idea is to ensure that those of lower social position and with greater need can benefit more than advantaged persons”

14. How equity focused is this quote when considering defining a topic?

1 = Not at all equity focused	2 = Slightly equity focused	3 = Neither equity focused nor unfocused	4= Equity focused	5 = Highly equity focused
○	○	○	○	○

15. Do you think that this is a good example of a highly equity focused quotation? Do you have another example in mind that might be better?

Equity in guideline development handbook - Domain 1

Quotation 2 - Defining a topic

“Considerations must include the link between the topic and national health-care priorities, and/or the relevance of the guideline.

6.2.1.1 *Burden of disease in Estonia*- It is important to consider the size of the patient/target group(s) affected by the disease or condition in Estonia (morbidity, prevalence, mortality, etc.).- The impact of the disease or condition on the Estonian health and social care system should also be taken into account.

6.2.1.2 *Differences in practice and/or health outcomes and/or costs*- Significant differences exist in practices and between/within patient groups (including subgroups); by health-care providers and/or different levels of care (e.g. primary care versus specialist medical care); in different regions of Estonia; or by different cost categories (medicinal products, inpatient treatment, etc.).- Differences also exist between Estonian and international practices.

6.2.1.3 *Expected impacts on patient health indicators and/or use of resources*- Anticipated impacts include:- modernizing current practices;- introduction of new interventions (including diagnostic and other tests and health-care services);- availability of new evidence-based practices, possibly altering current practices;- more efficient use of resources.”

16. How equity focused is this quote when considering defining a topic?

1 = Not at all equity focused	2 = Slightly equity focused	3 = Neither equity focused nor unfocused	4= Equity focused	5 = Highly equity focused
○	○	○	○	○

17. Do you think that this is a good example of a highly equity focused quotation? Do you have another example in mind that might be better?

Equity in guideline development handbook - Domain 1

Quotation 3 - Determining the scope

"During the development of the scope, it is important to consider and assess any health equality and equity issues to establish:

- whether there is any risk of unlawful discrimination arising from the guideline
- whether the guideline offers any opportunity for advancing equality or reducing inequalities
- whether there might need to be reasonable adjustments to a recommendation to avoid putting any group of people covered by the scope at substantial disadvantage
- whether and to what extend particular equality issues should be included in the scope"

18. How equity focused is this quote when considering determining the scope?

1 = Not at all equity focused	2 = Slightly equity focused	3 = Neither equity focused nor unfocused	4= Equity focused	5 = Highly equity focused
○	○	○	○	○

19. Do you think that this is a good example of a highly equity focused quotation? Do you have another example in mind that might be better?

Quotation 4 - Determining the scope

“**Determine Guideline Scope**

Affected Population: Developers might categorize affected populations by age (e.g., newborn babies), behavior (e.g., men who have sex with men), geography (e.g., populations in states in the Pacific Northwest), occupation (e.g., health care workers) or other criteria.

Intended Audience: Public health guidelines often have diverse audiences (e.g., practitioners, policy makers, health care businesses, and government agencies); therefore, they will need to balance the information according to multiple needs.

Guideline Setting: Settings might include doctors or dental offices, hospitals, nursing homes, day care facilities, schools, colleges, workplaces, pharmacies, supermarkets, or the community at large. Settings might be narrowed by the populations they serve. For example, school-based guidelines might focus on schools in low-income areas, which could be determined by factors such as the proportion of children who are eligible for free school lunches”

20. How equity focused is this quote when considering determining the scope?

1 = Not at all equity focused	2 = Slightly equity focused	3 = Neither equity focused nor unfocused	4= Equity focused	5 = Highly equity focused
○	○	○	○	○

21. Do you think that this is a good example of a highly equity focused quotation? Do you have another example in mind that might be better?

Equity in guideline development handbook - Domain 1

Quotation 5 - Identifying existing guidelines, adapting, and adopting

"**Adopting or adapting a guideline**: An 'equity lens' is a series of structured questions focusing on the visibility of equity, inequity and socioeconomic determinants, can be used to assess whether existing guidelines have explicitly considered the needs and values of disadvantaged populations."

22. How equity focused is this quote when considering identifying existing guidelines, adapting, and adopting

1 = Not at all equity focused	2 = Slightly equity focused	3 = Neither equity focused nor unfocused	4= Equity focused	5 = Highly equity focused
○	○	○	○	○

23. Do you think that this is a good example of a highly equity focused quotation? Do you have another example in mind that might be better?

Equity in guideline development handbook - Domain 1

Quotation 6- Identifying existing guidelines, adapting, and adopting

"5.4.3.2 Adaptation, implementation and evaluation (Chapter 13)

When developing guidelines, clear references to health equity, the social determinants of health, gender equality and relevant international human rights standards and principles, should be included in the sections on implementation, monitoring and evaluation. The interventions recommended in a guideline pose the risk of increasing rather than reducing inequities, depending on how they are implemented. For example, marginalized population groups might be ignored or strategies that make them the last to benefit might be used. Important gender differences might not be considered or unintended effects on certain subpopulations or population strata might occur. Such situations arise because decisions governing implementation are not explicitly related to equity. For instance, vaccination coverage targets might be achieved without benefit to the poorest and most disadvantaged groups. WHO staff have a responsibility to monitor and evaluate how recommendations affect equity, human rights, gender equality and the advancement of the highest attainable standard of health, and how health services and programmes implement the recommended interventions."

24. How equity focused is this quote when considering identifying existing guidelines, adapting, and adopting

1 = Not at all equity focused	2 = Slightly equity focused	3 = Neither equity focused nor unfocused	4= Equity focused	5 = Highly equity focused
○	○	○	○	○

25. Do you think that this is a good example of a highly equity focused quotation? Do you have another example in mind that might be better?

Thank you!

Thank you for participating in this survey! Your feedback will help us further develop the tool!

Equity in guideline development handbook - Domain 2

Informed consent

We (Ramaa Chitale, doctoral student, MPH, and Emily Smith, Associate Professor, ScD) are inviting you to take this survey for research. Participation is completely voluntary and there are no negative consequences if you do not participate in this survey. If you start the survey, you can stop at any time without completing it. At the end of this survey, we may ask you to participate in a second round of expert feedback, which involves an online Zoom group discussion. This is also voluntary. You are free to accept or decline participation in this follow up online Zoom discussion.

What is the purpose of this research?
The purpose of this study is to elicit expert feedback on a new tool that assesses equity considerations in guideline development handbooks for public health/clinical practice guidelines.

What will I do?
There are two types of questions for this survey. First read items from a new tool developed for the purpose of this study. Second, read a quotation from a commonly referenced guideline development handbook. There are 3 to 4 questions about each item in the tool. The survey will take about 45 min – 1 hour.

After this survey, you may be asked to participate in a second round to further elicit expert feedback on the new tool. The second round will be an online Zoom group discussion, which will last 1-2 hours.

Risks

- You might disagree with the way your organization handles equity in guideline development. However, none of these questions ask you about your role in guideline development. Instead, all questions are about your own opinions.
- Online data being hacked or intercepted: any time you share information online there are risks. We are using Survey Monkey, an online survey platform and George Washington University partnered Zoom account, and George Washington University Box account, but we cannot eliminate this risk entirely.
- Breach of confidentiality: there is a chance that your data could be seen by someone who is not meant to. We are minimizing the risk in the following ways:

- We will store all electronic data on a password protected encrypted computer.
- We will keep your identifying information separate from the research data, but we will link it to you. We will destroy this link after we are finished collecting and analyzing the data.
- If you participate in the second round of the survey, we will destroy the recording after collecting and analyzing the data from the online discussion.

Possible benefits: There is no direct benefit for you. However, your contributions will help to create a tool that may help organizations assess their own documents and understand the extent to which they do/do not consider health equity. In the future, these organizations may update their guideline development handbooks based on the discussions about the assessment tool.

Estimated number of participants: For the first round of the study, we will invite up to 40 participants for the online survey. We will then invite 20 participants of these original 40 back to the online Zoom group discussion.

How long will it take? This survey should take you no more than 1 hour. If you participate in the second round of expert feedback, this online discussion should take no more than 2 hours.

Costs: It will not cost you anything to participate in this study.

Compensation: You will not receive any compensation for participating in this study.

Future research: Your data will not be used or shared with any researchers or used for other future studies.

Confidentiality and data security: We collect the following information: name, email address, and job category. We also ask if you would like to be acknowledged in this work. This information is necessary to characterize participants.

Where will the data be stored: Data from the survey will be downloaded from SurveyMonkey (an online survey platform) and stored on the researcher's George Washington University Box account. For the second round of expert feedback (group discussion), researchers will use a George Washington University Zoom account to record the meeting, and store this recording on the George Washington University Zoom platform.

How long will it be kept? The Zoom recording will be destroyed after 6 months. The survey data and meeting notes will be kept 6 years after analysis.

Who can see my data?

- We (the researchers) will have access to identifiable (with your name included) data. This is so that we can analyze the data and conduct the study.
- We may share our findings in publications or presentations. If we do, results will be aggregated, with no identifiable information. If we quote you, we will use pseudonyms (fake names).

Questions about research, complaints, or problems: Contact Ramaa Chitale, rchitale@gwmail.gwu.edu 202-413-1712 or Emily Smith emilysmith@gwmail.gwu.edu 202-994-3589

Questions about your rights as a research participant, complaints, or problems: Contact George Washington University IRB (Institutional Review Board) at 202-994-2715 email: ohriirb@gwu.edu

Agreement to participate

Your participation is completely voluntary, and you can withdraw at any time. To take the survey you must be:

- At least 18 years old

If you meet the criteria and would like to take the survey, click the button below to start.

Equity in guideline development handbook - Domain 2

General information

* 1. Name

2. Current employer or academic institution

3. Which of the following describes your experience (select all that apply)?

- [ ] Guideline development panel member in the past or present
- [ ] Guideline development methodologist
- [ ] Health equity researcher with publication(s) about guidelines
- [ ] Guideline development handbook contributor (either helped develop a chapter or the entire guideline development handbook)
- [ ] Current student or postdoc conducting work either on health equity or guideline development
- [ ] Other (please specify)

* 4. Are you willing to be acknowledged in this dissertation and the accompanying peer reviewed manuscript?

- Yes
- No
- Not sure, please contact me to discuss further

Equity in guideline development handbook - Domain 2

Instructions for assessment

In the next **3** questions, I will show you a portion of the tool on Identifying the guideline group (forming guideline development group, involving stakeholders, managing conflicts of interest). If you would like to see the entire draft tool, please document attached to the email.

1) Read the “essential element” name, definition, and how to rate the guideline development handbook text.

a. The term “essential element” refers to methodological components that scholars generally agree are part of the guideline development process. <u>You will only see 3 “essential elements,” but a full list of all essential elements is available in the draft tool.</u>

2) Rate how equity focused you think the criteria are and provide additional feedback. Consider the criteria together. Criteria detail explicit language or subject matter that an assessor should seek when assessing the guideline development handbook.

****Remember, this tool is not for guidelines themselves, but for the guideline development handbooks that describe guideline development methodology.**

Essential element #4

Essential element #4 – Forming guideline development group

Essential element definition: Describes selection of the guideline development group, their roles, and responsibilities. Guideline development committees typically include a chair, a literature review group, voting members, etc.

How to rate the handbook– look for discussion of:

Criteria:

1. Involving stakeholders throughout the guideline process who are EITHER:
   a. Members of the affected subgroup and representatives OR, if this is not possible (e.g. in instances involving children or people with intellectual disabilities);
   b. Experts on a topic relevant to the subgroups (e.g., experts on inequities in service delivery, epidemiologist, economist etc.).
2. Defining when stakeholders will participate in guideline development process (e.g. voting, as subcommittee members, etc.)
3. Ensuring that the chair understands health equity and encourages participation from entire committee
4. Creating diversity within the guideline development group (e.g. balance of sex, race, expertise etc.)

5. Consider these criteria all together. If you were to assess a guideline development handbook for equity inclusion, how relevant are the listed criteria?

1 = Clearly not relevant	2	3	4	5	6	7	8	9 = Clearly relevant
○	○	○	○	○	○	○	○	○

6. Do you have feedback on additional criteria?

7. Do you have suggestions to improve the clarity of these criteria?

Equity in guideline development handbook - Domain 2

Essential element #5

Essential element #5 – Involving stakeholders

Essential element definition: Engaging people who are not part of the guideline development group but are affected by the guideline (e.g. target audience of users). Stakeholders often referred to as "consumers" in many guideline development handbooks

How to rate the handbook – look for discussion of:

Criteria

1. Recruiting and engaging representatives from relevant subgroups throughout the guideline development process.
   a. OR if it is not possible (e.g. guidelines involving children), suggestions of alternatives like discussions with other stakeholders (i.e. parents, caregivers, experts).
   b. OR if these alternatives are still not possible, the handbook suggests systematic review of previous data, etc.
2. Describing structured format for eliciting feedback and participation from stakeholders. For example, structured or semi-structured interviews with stakeholders, systematic reviews of qualitative/quantitative data, etc.
3. Offer training for stakeholders contributing to guideline development.

8. Consider these criteria all together. If you were to assess a guideline development handbook for equity inclusion, how relevant are the listed criteria?

1 = Clearly not relevant	2	3	4	5	6	7	8	9 = Clearly relevant
○	○	○	○	○	○	○	○	○

9. Do you have feedback on additional criteria?

10. Do you have suggestions to improve the clarity of these criteria?

Equity in guideline development handbook - Domain 2

Essential element #6

Essential element #6 – Managing conflicts of interest

Essential element definition: Defining and managing potential conflicts of interest of guideline development group members.

How to rate the handbook– look for discussion of:

Criteria

1. Identifying conflict of interest as a source of potential bias that may impact health equity considerations.
2. Outlining methods to manage: 1) financial and 2) non-financial conflicts of interest.
3. Requiring action related to conflict-of-interest disclosure, for instance, specifying when and how a panelist may serve in leadership roles

11. Consider these criteria all together. If you were to assess a guideline development handbook for equity inclusion, how relevant are the listed criteria?

1 = Clearly not relevant	2	3	4	5	6	7	8	9 = Clearly relevant
◯	◯	◯	◯	◯	◯	◯	◯	◯

12. Do you have feedback on additional criteria?

13. Do you have suggestions to improve the clarity of these criteria?

Equity in guideline development handbook - Domain 2

Instructions for quotation questions

Examples of equity focused quotations from other guideline development handbooks may help organizations model their own language.

In the next **6** questions, I will show you a quotation from a guideline development handbook. These quotes are from the guidelines identifying guideline group portion (forming guideline development group, involving stakeholders, managing conflicts of interest) of several commonly referenced guideline development handbooks.

Read each quotation and provide feedback.

Please note that some of these quotations are long because I did not want to take them out of context. However, I recognize that this is not fully possible, and is a limitation of the survey format.

The goal of these next questions is to identify equity-focused language that would help a future tool user know what to generally look for.

Quotation 1 - Forming guideline development group

“Group membership should not only reflect diversity, including geographical, gender balance or cultural background, but should also include populations with where the health conditions are more prevalent…all guidelines groups should try to include at least one indigenous representative….”

14. How equity focused is this quote when considering forming guideline development group?

1 = Not at all equity focused	2 = Slightly equity focused	3 = Neither equity focused nor unfocused	4= Equity focused	5 = Highly equity focused
○	○	○	○	○

15. Do you think that this is a good example of a highly equity focused quotation? Do you have another example in mind that might be better?

Equity in guideline development handbook - Domain 2

Quotation 2 - Forming guideline development group

“The Panel must represent a balance of the various health-care levels (primary care, hospital care, nursing care) according to the topic. It monitors the regional representation of experts and involves health professionals whose work will be most affected by the guideline. Panel members represent their own views and not those of organizations, although they may be recruited from or suggested by these organizations. The optimal Panel size is 8–10 members, but depending on the guideline topic and target group, it may be necessary to involve fewer or more members and to invite consultants for their input on individual issues.”

16. How equity focused is this quote when considering forming guideline development group?

1 = Not at all equity focused	2 = Slightly equity focused	3 = Neither equity focused nor unfocused	4= Equity focused	5 = Highly equity focused
◯	◯	◯	◯	◯

17. Do you think that this is a good example of a highly equity focused quotation? Do you have another example in mind that might be better?

Equity in guideline development handbook - Domain 2

Quotation 3 - Involving stakeholders

“Some approaches to consider include:

- formal, structured activities (e.g., interviews, focus groups, consensus methods)
- informal methods (e.g., project or advisory meetings, feedback on documents, public consultations
- modes of involvement (e.g., face-to-face, online, video-/teleconference, email)
- timing of involvement (e.g., one-ff, multistage, continuous)

You will need to be flexible and adapt to consumer needs for any approach you choose to take, especially if they live remotely or have ongoing health challenges”

18. How equity focused is this quote when considering involving stakeholders?

1 = Not at all equity focused	2 = Slightly equity focused	3 = Neither equity focused nor unfocused	4= Equity focused	5 = Highly equity focused
○	○	○	○	○

19. Do you think that this is a good example of a highly equity focused quotation? Do you have another example in mind that might be better?

Equity in guideline development handbook - Domain 2

Quotation 4 - Involving stakeholders

“Stakeholder engagement in guideline development enhances the relevance of the guidelines, creates a sense of ownership among stakeholders, and raises awareness about the project, ultimately facilitating acceptance, implementation, and adherence. It also contributes to the accountability and legitimacy of the guideline developers. Stakeholder engagement requires careful planning in terms of whom to engage, at which steps of the guideline development process, at what level of engagement, and using which mode. The following sections address these four questions. In addition, practical guidance on facilitators for stakeholder engagement, reporting on stakeholder engagement, and evaluation of stakeholder engagement are referenced.”

20. How equity focused is this quote when considering involving stakeholders?

1 = Not at all equity focused	2 = Slightly equity focused	3 = Neither equity focused nor unfocused	4= Equity focused	5 = Highly equity focused
○	○	○	○	○

21. Do you think that this is a good example of a highly equity focused quotation? Do you have another example in mind that might be better?

Equity in guideline development handbook - Domain 2

Quotation 5 - Managing conflicts of interest

“Can the interest consistently produce bias?

Consider whether the interest would produce a consistent direction of bias in the context of guideline development, such as if financial sponsorship were to influence decisions consistently in favor of an intervention or product preferred by the sponsor”

22. How equity focused is this quote when considering guideline managing conflicts of interest?

1 = Not at all equity focused	2 = Slightly equity focused	3 = Neither equity focused nor unfocused	4= Equity focused	5 = Highly equity focused
○	○	○	○	○

23. Do you think that this is a good example of a highly equity focused quotation? Do you have another example in mind that might be better?

Equity in guideline development handbook - Domain 2

Quotation 6- Managing conflicts of interest

Types of COI:

“Intellectual or academic - occurs when a person or a professional group is jeopardized or enhanced by a guideline recommendation. Guyatt defined this as ‘‘academic activities that create the potential for an attachment to a specific point of view that could unduly affect an individual’s judgment about a specific recommendation’ [Guyatt G et al. 2010]. Examples include having published a scientific paper on the topic, having received grant support related to the guideline, having personal beliefs related to the topic that may lead to biased writing and publishing, being a chair or member of another guideline committee relevant to the topic, involvement in an advocacy group that stands to benefit from recommendations, being a member of a lobbying or advocacy organization related to the topic, or having family members with the condition addressed by the CPG.”

24. How equity focused is this quote when considering managing conflicts of interest?

1 = Not at all equity focused	2 = Slightly equity focused	3 = Neither equity focused nor unfocused	4= Equity focused	5 = Highly equity focused
○	○	○	○	○

25. Do you think that this is a good example of a highly equity focused quotation? Do you have another example in mind that might be better?

Thank you!

Thank you for participating in this survey! Your feedback will help us further develop the tool!

Informed consent

We (Ramaa Chitale, doctoral student, MPH, and Emily Smith, Associate Professor, ScD) are inviting you to take this survey for research. Participation is completely voluntary and there are no negative consequences if you do not participate in this survey. If you start the survey, you can stop at any time without completing it. At the end of this survey, we may ask you to participate in a second round of expert feedback, which involves an online Zoom group discussion. This is also voluntary. You are free to accept or decline participation in this follow up online Zoom discussion.

What is the purpose of this research?
The purpose of this study is to elicit expert feedback on a new tool that assesses equity considerations in guideline development handbooks for public health/clinical practice guidelines.

What will I do?
There are two types of questions for this survey. First read items from a new tool developed for the purpose of this study. Second, read a quotation from a commonly referenced guideline development handbook. There are 3 to 4 questions about each item in the tool. The survey will take about 45 min – 1 hour.

After this survey, you may be asked to participate in a second round to further elicit expert feedback on the new tool. The second round will be an online Zoom group discussion, which will last 1-2 hours.

Risks

- You might disagree with the way your organization handles equity in guideline development. However, none of these questions ask you about your role in guideline development. Instead, all questions are about your own opinions.
- Online data being hacked or intercepted: any time you share information online there are risks. We are using Survey Monkey, an online survey platform and George Washington University partnered Zoom account, and George Washington University Box account, but we cannot eliminate this risk entirely.
- Breach of confidentiality: there is a chance that your data could be seen by someone who is not meant to. We are minimizing the risk in the following ways:

- We will store all electronic data on a password protected encrypted computer.
- We will keep your identifying information separate from the research data, but we will link it to you. We will destroy this link after we are finished collecting and analyzing the data.
- If you participate in the second round of the survey, we will destroy the recording after collecting and analyzing the data from the online discussion.

Possible benefits: There is no direct benefit for you. However, your contributions will help to create a tool that may help organizations assess their own documents and understand the extent to which they do/do not consider health equity. In the future, these organizations may update their guideline development handbooks based on the discussions about the assessment tool.

Estimated number of participants: For the first round of the study, we will invite up to 40 participants for the online survey. We will then invite 20 participants of these original 40 back to the online Zoom group discussion.

How long will it take? This survey should take you no more than 1 hour. If you participate in the second round of expert feedback, this online discussion should take no more than 2 hours.

Costs: It will not cost you anything to participate in this study.

Compensation: You will not receive any compensation for participating in this study.

Future research: Your data will not be used or shared with any researchers or used for other future studies.

Confidentiality and data security: We collect the following information: name, email address, and job category. We also ask if you would like to be acknowledged in this work. This information is necessary to characterize participants.

Where will the data be stored: Data from the survey will be downloaded from SurveyMonkey (an online survey platform) and stored on the researcher's George Washington University Box account. For the second round of expert feedback (group discussion), researchers will use a George Washington University Zoom account to record the meeting, and store this recording on the George Washington University Zoom platform.

How long will it be kept? The Zoom recording will be destroyed after 6 months. The survey data and meeting notes will be kept 6 years after analysis.

Who can see my data?

- We (the researchers) will have access to identifiable (with your name included) data. This is so that we can analyze the data and conduct the study.
- We may share our findings in publications or presentations. If we do, results will be aggregated, with no identifiable information. If we quote you, we will use pseudonyms (fake names).

Questions about research, complaints, or problems: Contact Ramaa Chitale, rchitale@gwmail.gwu.edu 202-413-1712 or Emily Smith emilysmith@gwmail.gwu.edu 202-994-3589

Questions about your rights as a research participant, complaints, or problems: Contact George Washington University IRB (Institutional Review Board) at 202-994-2715 email: ohriirb@gwu.edu

Agreement to participate

Your participation is completely voluntary, and you can withdraw at any time. To take the survey you must be:

- At least 18 years old

If you meet the criteria and would like to take the survey, click the button below to start.

Equity in guideline development handbook - Domain 3

General information

* 1. Name

2. Current employer or academic institution

3. Which of the following describes your experience (select all that apply)?

- [ ] Guideline development panel member in the past or present
- [ ] Guideline development methodologist
- [ ] Health equity researcher with publication(s) about guidelines
- [ ] Guideline development handbook contributor (either helped develop a chapter or the entire guideline development handbook)
- [ ] Current student or postdoc conducting work either on health equity or guideline development
- [ ] Other (please specify)

* 4. Are you willing to be acknowledged in this dissertation and the accompanying peer reviewed manuscript?

- ( ) Yes
- ( ) No
- ( ) Not sure, please contact me to discuss further

Equity in guideline development handbook - Domain 3

Instructions for assessment

In the next **6** questions, I will show you a portion of the tool on Gathering the evidence (establishing clinical questions, outcome consideration, systematic search for evidence and evidence inclusion, summarizing evidence, appraising evidence, conducting economic evaluation). If you would like to see the entire draft tool, please see the document attached to the email.

1) Read the "essential element" name, definition, and how to rate the guideline development handbook text.

a. The term "essential element" refers to methodological components that scholars generally agree are part of the guideline development process. You will only see 6 "essential elements," but a full list of all essential elements is available in the draft tool.

2) Rate how equity focused you think the criteria are and provide additional feedback. Consider the criteria together. Criteria detail explicit language or subject matter that an assessor should seek when assessing the guideline development handbook.

****Remember, this tool is not for guidelines themselves, but for the guideline development handbooks that describe guideline development methodology.**

Essential element #7

Essential element #7 – Establishing clinical questions

Essential element definition: Identify the key questions that a recommendation should address using a question framework. The most common frameworks are PICO (**P**opulation, **I**ntervention, **C**omparator, and **O**utcomes) and SPICE (**S**etting, **P**erspective, **I**ntervention/exposure/interest, **C**omparison, **E**valuation).

How to rate the handbook – look for discussion of:

Criteria:

1. Including questions on both the average health effects of an intervention and the distribution of health effects within a population
2. Considering health equity at each point of the question framework (e.g. PICO or SPICE). This means:
   a) Specifying a population of interest, especially subgroups stratified by any of the PROGRESS PLUS mnemonic
   b) Highlighting interventions aimed at subgroups of the population that reduce the health gradient
   c) Identifying outcomes that are important to disadvantaged subgroups within the population, either through stakeholder input or expert advice
   d) Identifying appropriate comparator group(s)

Abbreviation: PROGRESS PLUS= Place of residence, Race/ethnicity/culture/language, Occupation, Gender/sex, Religion, Education, Socioeconomic Status, Social capital, personal characteristics associated with discrimination (e.g. disability), features of relationships (smoking parents, excluded from school), time dependent relationships

5. Consider these criteria all together. If you were to assess a guideline development handbook for equity inclusion, how relevant are the listed criteria?

1 = Clearly not relevant	2	3	4	5	6	7	8	9 = Clearly relevant
○	○	○	○	○	○	○	○	○

6. Do you have feedback on additional criteria?

7. Do you have suggestions to improve the clarity of these criteria?

Equity in guideline development handbook - Domain 3

Essential element #8

Essential element #8 – Outcome consideration

Essential element definition: In the process of guideline development, incorporating how those impacted by the recommendation identify possible consequences (Schunemann et al, 2014)

How to rate the handbook– look for discussion of:

Criteria

1. Involving stakeholders when assessing interventions and identifying outcomes for guideline
2. Including stakeholder's intervention and outcomes priorities when searching for evidence.

8. Consider these criteria all together. If you were to assess a guideline development handbook for equity inclusion, how relevant are the listed criteria?

1 = Clearly not relevant	2	3	4	5	6	7	8	9 = Clearly relevant
○	○	○	○	○	○	○	○	○

9. Do you have feedback on additional criteria?

10. Do you have suggestions to improve the clarity of these criteria?

Essential element #9

Essential element #9 – Systematic search for evidence and evidence inclusion

Essential element definition: Creating an inclusion and exclusion criteria for evidence, study design, population, interventions, and comparators. Deciding how to identify and obtain evidence.

How to rate the handbook– look for discussion of:

Criteria

1. Searching multiple databases for relevant information
2. Searching non-English studies when appropriate (not applicable to all handbooks)
3. Using search terms with filters/combination of terms related to the subgroup of interest (for instance, include free text/subject headings for geographic locations)
4. Including a variety of study types (observational, qualitative, quantitative)
5. Drawing from other disciplines when needed to address health concerns of subgroups

11. Consider these criteria all together. If you were to assess a guideline development handbook for equity inclusion, how relevant are the listed criteria?

1 = Clearly not relevant	2	3	4	5	6	7	8	9 = Clearly relevant
○	○	○	○	○	○	○	○	○

12. Do you have feedback on additional criteria?

13. Do you have suggestions to improve the clarity of these criteria?

Equity in guideline development handbook - Domain 3

Essential element #10

Essential element #10 – Summarizing the evidence

Essential element definition: Describes synthesis of the evidence in the form of tables, charts, or brief narratives. May also include additional information that does not fit into all the tables.

How to rate the handbook – look for discussion of:

Criteria

1. Including health equity as an outcome in the summary of finding (SoF) table and any statements of insufficient evidence for impact on health equity
2. Presenting both absolute and relative measures of inequality (between relatively advantaged and relatively disadvantaged)
   a. Assess differences in baseline when presenting absolute measures and use appropriate reference groups

14. Consider these criteria all together. If you were to assess a guideline development handbook for equity inclusion, how relevant are the listed criteria?

1 = Clearly not relevant	2	3	4	5	6	7	8	9 = Clearly relevant
○	○	○	○	○	○	○	○	○

15. Do you have feedback on additional criteria?

16. Do you have suggestions to improve the clarity of these criteria?

Equity in guideline development handbook - Domain 3

Essential element #11

Essential element #11 – Appraising the evidence

Essential element definition: Assessing the confidence a developer can have by evaluating the "strength and quality" of that evidence. This might include structured approaches like GRADE which assign a "certainty of evidence" by PICO

How to rate the handbook – look for discussion of:

Criteria

1. Assessing indirectness of evidence using the GRADE approach
2. Acknowledging that certainty of evidence should not be downgraded for indirectness for differences within a population, unless "there are compelling reasons to anticipate differences in effect due to biology/physiology, sociocultural influences, or setting-specific resource issues that impact effectiveness or harms of the intervention" (Welch et al, 2017)
3. Guidance on assessing quality of evidence for qualitative studies (using GRADE-CERQual)

17. Consider these criteria all together. If you were to assess a guideline development handbook for equity inclusion, how relevant are the listed criteria?

1 = Clearly not relevant	2	3	4	5	6	7	8	9 = Clearly relevant
○	○	○	○	○	○	○	○	○

18. Do you have feedback on additional criteria?

19. Do you have suggestions to improve the clarity of these criteria?

Equity in guideline development handbook - Domain 3

Essential element #12

Essential element #12 – Conducting economic evaluation

Essential element definition: Identifying and analyzing additional economic information. Presenting cost and benefit.

How to rate the handbook– look for discussion of:

Criteria

1. Including methods to incorporate equity into guideline development during economic analysis phase. These methods might include equity weighting, extended or distributional cost-effectiveness analysis, economic analysis by subgroups, etc.

20. Consider these criteria all together. If you were to assess a guideline development handbook for equity inclusion, how relevant are the listed criteria?

1 = Clearly not relevant	2	3	4	5	6	7	8	9 = Clearly relevant
○	○	○	○	○	○	○	○	○

21. Do you have feedback on additional criteria?

22. Do you have suggestions to improve the clarity of these criteria?

Equity in guideline development handbook - Domain 3

Instructions for quotation questions

Examples of equity focused quotations from other guideline development handbooks may help organizations model their own language.

In the next **6** questions, I will show you a quotation from a guideline development handbook. These quotes are from the guidelines gathering the evidence (establishing clinical questions, outcomes consideration, systematic search for evidence and evidence inclusion, appraising the evidence, and conducting economic evaluation) of several commonly referenced guideline development handbooks.

Read each quotation and provide feedback.

Please note that some of these quotations are long because I did not want to take them out of context. However, I recognize that this is not fully possible, and is a limitation of the survey format.

The goal of these next questions is to identify equity-focused language that would help a future tool user know what to generally look for.

Equity in guideline development handbook - Domain 3

Quotation 1 - Establishing clinical questions

"For each review question, factors that may affect the outcomes and effectiveness of an intervention, including any wider social factors that may affect health and any health inequalities, should be considered"

23. How equity focused is this quote when considering establishing clinical questions?

1 = Not at all equity focused	2 = Slightly equity focused	3 = Neither equity focused nor unfocused	4= Equity focused	5 = Highly equity focused
○	○	○	○	○

24. Do you think that this is a good example of a highly equity focused quotation? Do you have another example in mind that might be better?

Equity in guideline development handbook - Domain 3

Quotation 2 - Outcome consideration

“**Patient important outcomes** should be explicitly considered along with more narrowly defined clinically important outcomes. It is particularly important to include any potential harm associated with the intervention under review so that a balanced view can be taken at the considered judgement stage."

25. How equity focused is this quote when considering outcome consideration?

1 = Not at all equity focused	2 = Slightly equity focused	3 = Neither equity focused nor unfocused	4= Equity focused	5 = Highly equity focused
○	○	○	○	○

26. Do you think that this is a good example of a highly equity focused quotation? Do you have another example in mind that might be better?

Quotation 3 - Systematic search for evidence and evidence inclusion

“Databases relevant to these groups should be identified and searched, such as the Informit Indigenous Collection and the Aboriginal and Torres Strait islander Health Bibliography (Aboriginal and Torres Strait Islander Health, compiled by the Australian Indigenous Health Infonet), both of which provide searchable databases of literature about Aboriginal and Torres Strait Islander health”

27. How equity focused is this quote when considering systematic search for evidence and evidence inclusion?

1 = Not at all equity focused	2 = Slightly equity focused	3 = Neither equity focused nor unfocused	4= Equity focused	5 = Highly equity focused
○	○	○	○	○

28. Do you think that this is a good example of a highly equity focused quotation? Do you have another example in mind that might be better?

Equity in guideline development handbook - Domain 3

Quotation 4 - Summarizing the evidence

"One of the key objectives for evidence synthesis is to explore the reasons for difference in observed effects and to identify any populations or interventions/exposure categories that are associated with these differences. This can be a critical area of investigation used to inform a guideline's recommendations to support specific actions, for different populations. It is especially relevant to considerations of equity."

29. How equity focused is this quote when considering summarizing the evidence?

1 = Not at all equity focused	2 = Slightly equity focused	3 = Neither equity focused nor unfocused	4= Equity focused	5 = Highly equity focused
○	○	○	○	○

30. Do you think that this is a good example of a highly equity focused quotation? Do you have another example in mind that might be better?

Quotation 5 - Appraising the evidence

"The concept of indirectness refers to whether the evidence available, including the population, comparisons and outcomes measured, directly and completely answers the question proposed by the guideline…examples of important indirectness might include:

- Randomized trials of narrow segments of the population such as only participants with relatively mind illnesses or only adult,
- Studies conducted in high-income, urban settings rather than including rural or low-income settings…"

31. How equity focused is this quote when considering appraising the evidence?

1 = Not at all equity focused	2 = Slightly equity focused	3 = Neither equity focused nor unfocused	4= Equity focused	5 = Highly equity focused
○	○	○	○	○

32. Do you think that this is a good example of a highly equity focused quotation? Do you have another example in mind that might be better?

Equity in guideline development handbook - Domain 3

Quotation 6- Conducting economic evaluation

“to support our commitment to addressing health inequalities, we have commissioned a prototype tool to explore the approach of providing quantitative estimates of the impact of NICE recommendations on health inequalities. The tool uses distributional cost-effectiveness analysis to model changes in health inequalities between 5 socioeconomic groups in England based on the neighborhood index of multiple deprivation”

33. How equity focused is this quote when considering conducting economic evaluation?

1 = Not at all equity focused	2 = Slightly equity focused	3 = Neither equity focused nor unfocused	4= Equity focused	5 = Highly equity focused
○	○	○	○	○

34. Do you think that this is a good example of a highly equity focused quotation? Do you have another example in mind that might be better?

Thank you!

Thank you for participating in this survey! Your feedback will help us further develop the tool!

Equity in guideline development handbook - Domain 4

Informed consent

We (Ramaa Chitale, doctoral student, MPH, and Emily Smith, Associate Professor, ScD) are inviting you to take this survey for research. Participation is completely voluntary and there are no negative consequences if you do not participate in this survey. If you start the survey, you can stop at any time without completing it. At the end of this survey, we may ask you to participate in a second round of expert feedback, which involves an online Zoom group discussion. This is also voluntary. You are free to accept or decline participation in this follow up online Zoom discussion.

What is the purpose of this research?
The purpose of this study is to elicit expert feedback on a new tool that assesses equity considerations in guideline development handbooks for public health/clinical practice guidelines.

What will I do?
There are two types of questions for this survey. First read items from a new tool developed for the purpose of this study. Second, read a quotation from a commonly referenced guideline development handbook. There are 3 to 4 questions about each item in the tool. The survey will take about 45 min – 1 hour.

After this survey, you may be asked to participate in a second round to further elicit expert feedback on the new tool. The second round will be an online Zoom group discussion, which will last 1-2 hours.

Risks

- You might disagree with the way your organization handles equity in guideline development. However, none of these questions ask you about your role in guideline development. Instead, all questions are about your own opinions.
- Online data being hacked or intercepted: any time you share information online there are risks. We are using Survey Monkey, an online survey platform and George Washington University partnered Zoom account, and George Washington University Box account, but we cannot eliminate this risk entirely.
- Breach of confidentiality: there is a chance that your data could be seen by someone who is not meant to. We are minimizing the risk in the following ways:

- We will store all electronic data on a password protected encrypted computer.
- We will keep your identifying information separate from the research data, but we will link it to you. We will destroy this link after we are finished collecting and analyzing the data.
- If you participate in the second round of the survey, we will destroy the recording after collecting and analyzing the data from the online discussion.

Possible benefits: There is no direct benefit for you. However, your contributions will help to create a tool that may help organizations assess their own documents and understand the extent to which they do/do not consider health equity. In the future, these organizations may update their guideline development handbooks based on the discussions about the assessment tool.

Estimated number of participants: For the first round of the study, we will invite up to 40 participants for the online survey. We will then invite 20 participants of these original 40 back to the online Zoom group discussion.

How long will it take? This survey should take you no more than 1 hour. If you participate in the second round of expert feedback, this online discussion should take no more than 2 hours.

Costs: It will not cost you anything to participate in this study.

Compensation: You will not receive any compensation for participating in this study.

Future research: Your data will not be used or shared with any researchers or used for other future studies.

Confidentiality and data security: We collect the following information: name, email address, and job category. We also ask if you would like to be acknowledged in this work. This information is necessary to characterize participants.

Where will the data be stored: Data from the survey will be downloaded from SurveyMonkey (an online survey platform) and stored on the researcher's George Washington University Box account. For the second round of expert feedback (group discussion), researchers will use a George Washington University Zoom account to record the meeting, and store this recording on the George Washington University Zoom platform.

How long will it be kept? The Zoom recording will be destroyed after 6

months. The survey data and meeting notes will be kept 6 years after analysis.

Who can see my data?

- We (the researchers) will have access to identifiable (with your name included) data. This is so that we can analyze the data and conduct the study.
- We may share our findings in publications or presentations. If we do, results will be aggregated, with no identifiable information. If we quote you, we will use pseudonyms (fake names).

Questions about research, complaints, or problems: Contact Ramaa Chitale, rchitale@gwmail.gwu.edu 202-413-1712 or Emily Smith emilysmith@gwmail.gwu.edu 202-994-3589

Questions about your rights as a research participant, complaints, or problems: Contact George Washington University IRB (Institutional Review Board) at 202-994-2715 email: ohriirb@gwu.edu

Agreement to participate

Your participation is completely voluntary, and you can withdraw at any time. To take the survey you must be:

- At least 18 years old

If you meet the criteria and would like to take the survey, click the button below to start.

Equity in guideline development handbook - Domain 4

General information

* 1. Name

2. Current employer or academic institution

3. Which of the following describes your experience (select all that apply)?

- [ ] Guideline development panel member in the past or present
- [ ] Guideline development methodologist
- [ ] Health equity researcher with publication(s) about guidelines
- [ ] Guideline development handbook contributor (either helped develop a chapter or the entire guideline development handbook)
- [ ] Current student or postdoc conducting work either on health equity or guideline development
- [ ] Other (please specify)

* 4. Are you willing to be acknowledged in this dissertation and the accompanying peer reviewed manuscript?

- ( ) Yes
- ( ) No
- ( ) Not sure, please contact me to discuss further

Equity in guideline development handbook - Domain 4

Instructions for assessment

In the next **3** questions, I will show you a portion of the tool on drafting and review (creating recommendations, wording and considering ethics, and reporting and consulting experts) If you would like to see the entire draft tool, please see the attached document.

1) Read the “essential element” name, definition, and how to rate the guideline development handbook text.

a. The term “essential element” refers to methodological components that scholars generally agree are part of the guideline development process. You will only see 3 “essential elements,” but a full list of all essential elements is available in the draft tool.

2) Rate how equity focused you think the criteria are and provide additional feedback. Consider the criteria together. Criteria detail explicit language or subject matter that an assessor should seek when assessing the guideline development handbook.

****Remember, this tool is not for guidelines themselves, but for the guideline development handbooks that describe guideline development methodology.**

Equity in guideline development handbook - Domain 4

Essential element #13

Essential element #13 – Creating recommendations

Essential element definition: Includes interpreting evidence and developing recommendations using a structured approach (like the Evidence to Decision framework). Strength of the recommendations refers to the judgement about "how confident a guideline panel is that the implementation of the recommendation exerts more desirable than undesirable consequences."

How to rate the handbook– look for discussion of:

Criteria:
1. Balancing benefits and harms (identifying potential unintended consequences) of proposed interventions to population subgroups
2. Creating equity-focused recommendations through consideration of interventions and their impacts on certain groups
3. Using an equity focused recommendations framework (e.g. WHO-INTEGRATE EtD framework)

Abbreviations: EtD= evidence to decision

5. Consider these criteria all together. If you were to assess a guideline development handbook for equity inclusion, how relevant are the listed criteria?

1 = Clearly not relevant	2	3	4	5	6	7	8	9 = Clearly relevant
○	○	○	○	○	○	○	○	○

6. Do you have feedback on additional criteria?

7. Do you have suggestions to improve the clarity of these criteria?

Equity in guideline development handbook - Domain 4

Essential element #14

Essential element #14 – Wording and considering ethics

Essential element definition: Has to do with the wording and syntax of recommendations as it relates to ethics including equity considerations

How to rate the handbook– look for discussion of:

Criteria

1. Avoiding language that may cause harm for certain population subgroups
2. Using specific language when defining population subgroups. This involves highlighting social determinants of health over simplifying terms based on race, gender, etc.
3. Translating the guideline into local language when relevant.

8. Consider these criteria all together. If you were to assess a guideline development handbook for equity inclusion, how relevant are the listed criteria?

1 = Clearly not relevant	2	3	4	5	6	7	8	9 = Clearly relevant
○	○	○	○	○	○	○	○	○

9. Do you have feedback on additional criteria?

10. Do you have suggestions to improve the clarity of these criteria?

Essential element #15

Essential element #15 – Reporting and consulting experts

Essential element definition: Refers to how a draft guideline will be assessed before implementation by stakeholders and members of the guideline review committee

How to rate the handbook– look for discussion of:

Criteria

1. Sharing final recommendations with stakeholders, and allow for comment
   a. Commenting might be through 1) public meeting, 2) submission for public consultation, or 3) posted on website for comment
2. Incorporating suggestions from stakeholders into final version of recommendations

11. Consider these criteria all together. If you were to assess a guideline development handbook for equity inclusion, how relevant are the listed criteria?

1 = Clearly not relevant	2	3	4	5	6	7	8	9 = Clearly relevant
○	○	○	○	○	○	○	○	○

12. Do you have feedback on additional criteria?

13. Do you have suggestions to improve the clarity of these criteria?

Equity in guideline development handbook - Domain 4

Instructions for quotation questions

Examples of equity focused quotations from other guideline development handbooks may help organizations model their own language.

In the next **6** questions, I will show you a quotation from a guideline development handbook. These quotes are from the guidelines drafting and review portion (creating recommendations, wording and considering ethics, and reporting and consulting experts) of several commonly referenced guideline development handbooks.

Read each quotation and provide feedback.

Please note that some of these quotations are long because I did not want to take them out of context. However, I recognize that this is not fully possible, and is a limitation of the survey format.

The goal of these next questions is to identify equity-focused language that would help a future tool user know what to generally look for.

Equity in guideline development handbook - Domain 4

Quotation 1 - Creating recommendations

“The committee should also assess the extent to which recommendations may impact on health inequalities. This needs to be made clear, regardless of whether the recommendation is aimed at the whole population, specific subgroups or a combination of both”)

14. How equity focused is this quote when considering creating recommendations?

1 = Not at all equity focused	2 = Slightly equity focused	3 = Neither equity focused nor unfocused	4= Equity focused	5 = Highly equity focused
○	○	○	○	○

15. Do you think that this is a good example of a highly equity focused quotation? Do you have another example in mind that might be better?

Equity in guideline development handbook - Domain 4

Quotation 2 - Creating recommendations

“5.4.2.3 Developing recommendations (Chapter 10)

The effect of an intervention on equity is one of the factors that determine the strength of a recommendation: if an intervention is likely to reduce health inequities, a strong recommendation may be warranted, provided it is justifiable in light of other factors. On the other hand, if equity is a key consideration and evidence on the intervention’s potential effects on equity is sparse, a conditional recommendation may be appropriate. In the latter case particularly, the guideline should outline the key gaps in knowledge and provide an agenda for future research. Evaluation and monitoring of the impact of recommendations that potentially affect inequities are also critically important and should be articulated in the guideline document.”

16. How equity focused is this quote when considering creating recommendations?

1 = Not at all equity focused	2 = Slightly equity focused	3 = Neither equity focused nor unfocused	4= Equity focused	5 = Highly equity focused
○	○	○	○	○

17. Do you think that this is a good example of a highly equity focused quotation? Do you have another example in mind that might be better?

Equity in guideline development handbook - Domain 4

Quotation 3 - Wording and considering ethics

“Avoid labelling people. Conditions describe what the person has, not what the person is. Diseases are treated, not people. Diseases, not people, respond to treatment. Conditions, not people, are monitored. People are not unsuitable for treatments: treatments are unsuitable for them. People have diseases, they do not suffer from them”

18. How equity focused is this quote when considering wording and considering ethics?

1 = Not at all equity focused	2 = Slightly equity focused	3 = Neither equity focused nor unfocused	4= Equity focused	5 = Highly equity focused
○	○	○	○	○

19. Do you think that this is a good example of a highly equity focused quotation? Do you have another example in mind that might be better?

Equity in guideline development handbook - Domain 4

Quotation 4 - Wording and considering ethics

"Throughout the process careful attention should be paid to the use of language, the selection of case examples, the expressions used to refer to population groups, the choice of photographs, etc. Setting the ground rules up front is much easier than trying to modify a document that is ready for layout. In translations of guidelines, gender-sensitive and non-stigmatizing language should always be employed."

20. How equity focused is this quote when considering guideline wording and considering ethics?

1 = Not at all equity focused	2 = Slightly equity focused	3 = Neither equity focused nor unfocused	4= Equity focused	5 = Highly equity focused
○	○	○	○	○

21. Do you think that this is a good example of a highly equity focused quotation? Do you have another example in mind that might be better?

Equity in guideline development handbook - Domain 4

Quotation 5 - Reporting and consulting experts

“Developers must take the following key points into account when responding to comments from registered stakeholders:

- Each comment must be acknowledged and answered as directly, full and with as much information as possible…
- For a draft guideline, the committee must consider whether changes to the guideline are needed as a result of consultation comments; any changes to the guideline must be agreed by the committee before publication.
- If changes are made to a guideline as a result of a consultation comment, this must be made clear in the response to the comment. If no changes have been made, it should be clear from the response why not...”

22. How equity focused is this quote when considering reporting and consulting experts?

1 = Not at all equity focused	2 = Slightly equity focused	3 = Neither equity focused nor unfocused	4= Equity focused	5 = Highly equity focused
○	○	○	○	○

23. Do you think that this is a good example of a highly equity focused quotation? Do you have another example in mind that might be better?

Quotation 6- Reporting and consulting experts

"Public consultation methods can be either open, targeted or a combination of the two (GIN Public 2012). By inviting specific stakeholders to comment, targeted consultation methods enable feedback to be sought in a relatively controlled manner; however, this also runs the risk of important viewpoints being overlooked. Open consultation is more transparent and can capture views from individuals or groups that might not otherwise have been planning or expected to engage."

24. How equity focused is this quote when reporting and consulting experts?

1 = Not at all equity focused	2 = Slightly equity focused	3 = Neither equity focused nor unfocused	4= Equity focused	5 = Highly equity focused
○	○	○	○	○

25. Do you think that this is a good example of a highly equity focused quotation? Do you have another example in mind that might be better?

Thank you!

Thank you for participating in this survey! Your feedback will help us further develop the tool!

Equity in guideline development handbooks - Domain 5

Informed consent

We (Ramaa Chitale, doctoral student, MPH, and Emily Smith, Associate Professor, ScD) are inviting you to take this survey for research. Participation is completely voluntary and there are no negative consequences if you do not participate in this survey. If you start the survey, you can stop at any time without completing it. At the end of this survey, we may ask you to participate in a second round of expert feedback, which involves an online Zoom group discussion. This is also voluntary. You are free to accept or decline participation in this follow up online Zoom discussion.

What is the purpose of this research?

The purpose of this study is to elicit expert feedback on a new tool that assesses equity considerations in guideline development handbooks for public health/clinical practice guidelines.

What will I do?

There are two types of questions for this survey. First read items from a new tool developed for the purpose of this study. Second, read a quotation from a commonly referenced guideline development handbook. There are 3 to 4 questions about each item in the tool. The survey will take about 45 min – 1 hour.

After this survey, you may be asked to participate in a second round to further elicit expert feedback on the new tool. The second round will be an online Zoom group discussion, which will last 1-2 hours.

Risks

- You might disagree with the way your organization handles equity in guideline development. However, none of these questions ask you about your role in guideline development. Instead, all questions are about your own opinions.
- Online data being hacked or intercepted: any time you share information online there are risks. We are using Survey Monkey, an online survey platform and George Washington University partnered Zoom account, and George Washington University Box account, but we cannot eliminate this risk entirely.
- Breach of confidentiality: there is a chance that your data could be seen by someone who is not meant to. We are minimizing the risk in the following ways:

- We will store all electronic data on a password protected encrypted computer.
- We will keep your identifying information separate from the research data, but we will link it to you. We will destroy this link after we are finished collecting and analyzing the data.
- If you participate in the second round of the survey, we will destroy the recording after collecting and analyzing the data from the online discussion.

Possible benefits: There is no direct benefit for you. However, your contributions will help to create a tool that may help organizations assess their own documents and understand the extent to which they do/do not consider health equity. In the future, these organizations may update their guideline development handbooks based on the discussions about the assessment tool.

Estimated number of participants: For the first round of the study, we will invite up to 40 participants for the online survey. We will then invite 20 participants of these original 40 back to the online Zoom group discussion.

How long will it take? This survey should take you no more than 1 hour. If you participate in the second round of expert feedback, this online discussion should take no more than 2 hours.

Costs: It will not cost you anything to participate in this study.

Compensation: You will not receive any compensation for participating in this study.

Future research: Your data will not be used or shared with any researchers or used for other future studies.

Confidentiality and data security: We collect the following information: name, email address, and job category. We also ask if you would like to be acknowledged in this work. This information is necessary to characterize participants.

Where will the data be stored: Data from the survey will be downloaded from SurveyMonkey (an online survey platform) and stored on the researcher's George Washington University Box account. For the second round of expert feedback (group discussion), researchers will use a George Washington University Zoom account to record the meeting, and store this recording on the George Washington University Zoom platform.

How long will it be kept? The Zoom recording will be destroyed after 6 months. The survey data and meeting notes will be kept 6 years after analysis.

Who can see my data?

- We (the researchers) will have access to identifiable (with your name included) data. This is so that we can analyze the data and conduct the study.
- We may share our findings in publications or presentations. If we do, results will be aggregated, with no identifiable information. If we quote you, we will use pseudonyms (fake names).

Questions about research, complaints, or problems: Contact Ramaa Chitale, rchitale@gwmail.gwu.edu 202-413-1712 or Emily Smith emilysmith@gwmail.gwu.edu 202-994-3589

Questions about your rights as a research participant, complaints, or problems: Contact George Washington University IRB (Institutional Review Board) at 202-994-2715 email: ohriirb@gwu.edu

Agreement to participate

Your participation is completely voluntary, and you can withdraw at any time. To take the survey you must be:

- At least 18 years old

If you meet the criteria and would like to take the survey, click the button below to start.

Equity in guideline development handbooks - Domain 5

General information

* 1. Name

2. Current employer or academic institution

3. Which of the following describes your experience (select all that apply)?

- [ ] Guideline development panel member in the past or present
- [ ] Guideline development methodologist
- [ ] Health equity researcher with publication(s) about guideline
- [ ] Guideline development handbook contributor (either helped develop a chapter or the entire guideline development handbook)
- [ ] Current student or postdoc conducting work either on health equity or guideline development
- [ ] Other (please specify)

* 4. Are you willing to be acknowledged in this dissertation and the accompanying peer reviewed manuscript?

- ( ) Yes
- ( ) No
- ( ) Not sure, please contact me to discuss further

Equity in guideline development handbooks - Domain 5

Instructions for assessment

In the next **3** questions, I will show you a portion of the tool on Dissemination, implementation, evaluation, and updating (dissemination and publications, implementing and evaluation, updating). If you would like to see the entire draft tool, please see the document attached to the email.

1) Read the “essential element” name, definition, and how to rate the guideline development handbook text.

a. The term “essential element” refers to methodological components that scholars generally agree are part of the guideline development process. <u>You will only see 3 “essential elements,” but a full list of all essential elements is available in the draft tool.</u>

2) Rate how equity focused you think the criteria are and provide additional feedback. Consider the criteria together. Criteria detail explicit language or subject matter that an assessor should seek when assessing the guideline development handbook.

**Remember, this tool is not for guidelines themselves, but for the guideline development handbooks that describe guideline development methodology.

Equity in guideline development handbooks - Domain 5

Essential element #16

Essential element #16 – Dissemination and publications

Essential element definition: Describes publication of guideline in different formats to ensure "relevant groups" are aware of their publication

How to rate the handbook – look for discussion of:

Criteria:

1. Creating multiple publication types of recommendations to ensure a wide range of outreach: this might include peer review publication, short guideline summary, technical report, lay versions , online or in print version
2. Allowing stakeholder comment on final guideline products to identify any problems with the guideline

5. Consider these criteria all together. If you were to assess a guideline development handbook for equity inclusion, how relevant are the listed criteria?

1 = Clearly not relevant	2	3	4	5	6	7	8	9 = Clearly relevant
○	○	○	○	○	○	○	○	○

6. Do you have feedback on additional criteria?

7. Do you have suggestions to improve the clarity of these criteria?

Equity in guideline development handbooks - Domain 5

Essential element #17

Essential element #17 – Implementation and evaluation

Essential element definition: Describes how guidelines are put into practice, including strategies so that "relevant groups are aware of the guidelines to enhance their uptake."

How to rate the handbook– look for discussion of:

Criteria

1. Creating tools/systems that monitor guideline uptake in population subgroups
2. Using indicators stratified by population subgroup characteristics to monitor health disparities or measure implementation within subgroup

8. Consider these criteria all together. If you were to assess a guideline development handbook for equity inclusion, how relevant are the listed criteria?

1 = Clearly not relevant	2	3	4	5	6	7	8	9 = Clearly relevant
○	○	○	○	○	○	○	○	○

9. Do you have feedback on additional criteria?

10. Do you have suggestions to improve the clarity of these criteria?

Essential element #18

Essential element #18 – Updating

Essential element definition: Describes how and when a guideline needs revision based on availability of new evidence or other factors

How to rate the handbook– look for discussion of:

Criteria

1. Monitoring new evidence on intervention that may impact subgroups of the population and present this evidence and include it in the update.
2. Including stakeholders in the entire updating process (either for an entire guideline or partial update). The updating process includes: forming groups to update guideline, identifying new evidence, assessment for the need to update, updating process, external review, and publication

11. Consider these criteria all together. If you were to assess a guideline development handbook for equity inclusion, how relevant are the listed criteria?

1 = Clearly not relevant	2	3	4	5	6	7	8	9 = Clearly relevant
○	○	○	○	○	○	○	○	○

12. Do you have feedback on additional criteria?

13. Do you have suggestions to improve the clarity of these criteria?

Equity in guideline development handbooks - Domain 5

Instructions for quotation questions

Examples of equity focused quotations from other guideline development handbooks may help organizations model their own language.

In the next **6** questions, I will show you a quotation from a guideline development handbook. These quotes are from the guidelines dissemination, implementation, evaluation, and updating portion (dissemination and publications, implementation and evaluation, and updating) of several commonly referenced guideline development handbooks.

Read each quotation and provide feedback.

Please note that some of these quotations are long because I did not want to take them out of context. However, I recognize that this is not fully possible, and is a limitation of the survey format.

The goal of these next questions is to identify equity-focused language that would help a future tool user know what to generally look for.

Equity in guideline development handbooks - Domain 5

Quotation 1 -Dissemination and publications

"NICE may use a range of different methods to raise awareness of the guideline. These include standard approaches such as:

- notifying registered stakeholders of publication
- publicizing the guideline through NICE's newsletters and alerts
- issuing press release as appropriate, posting news articles and blogs on the NICE website, using social media channels and promoting the guideline within NICE"

14. How equity focused is this quote when considering dissemination and publications?

1 = Not at all equity focused	2 = Slightly equity focused	3 = Neither equity focused nor unfocused	4= Equity focused	5 = Highly equity focused
○	○	○	○	○

15. Do you think that this is a good example of a highly equity focused quotation? Do you have another example in mind that might be better?

Equity in guideline development handbooks - Domain 5

Quotation 2 - Dissemination and publications

“Patients as champions for change

Patients are a powerful agent for change in the health service. Guidelines are published with an accompanying patient and carer version of the guideline and by being aware of a clinical guideline, patients can ask for their care to be in line with the latest recommendations. Making use of connections with patient groups and voluntary organisations also affords more opportunities to raise awareness of guidelines. Lay representatives on guideline development groups are supported to raise awareness at conferences and other events.”

16. How equity focused is this quote when considering dissemination and publications?

1 = Not at all equity focused	2 = Slightly equity focused	3 = Neither equity focused nor unfocused	4= Equity focused	5 = Highly equity focused
○	○	○	○	○

17. Do you think that this is a good example of a highly equity focused quotation? Do you have another example in mind that might be better?

Equity in guideline development handbooks - Domain 5

Quotation 3 - Implementation and evaluation

"Topic experts (including members of NICE's Expert Advisers Panel) are invited to participate in surveillance. They provide their views about how an event affects the recommendations, and their knowledge of recent developments in the topic area. If the response from topic experts is limited, or further specialist input is needed, we may seek input from other experts."

18. How equity focused is this quote when considering implementation and evaluation?

1 = Not at all equity focused	2 = Slightly equity focused	3 = Neither equity focused nor unfocused	4= Equity focused	5 = Highly equity focused
○	○	○	○	○

19. Do you think that this is a good example of a highly equity focused quotation? Do you have another example in mind that might be better?

Equity in guideline development handbooks - Domain 5

Quotation 4 - Implementation and evaluation

“2. Gather information about implementation throughout development

Discussions about guideline implementation may happen in the initial scoping phase of a guideline when evidence-practice or knowledge gaps are being identified. If a guideline already exists in this field, but is presumably out of date, the first question you should ask is whether it was implemented and, if so, whether it was implemented successfully. Evaluation data are rarely available for guidelines in Australia (NHMRC 2014) and so it is likely that you will need to gather this information through stakeholder consultation. In this initial phase of guideline development it is important to document any discussions or feedback, whether formal or informal, regarding implementation ideas to inform future activities.”

20. How equity focused is this quote when considering implementation and evaluation?

1 = Not at all equity focused	2 = Slightly equity focused	3 = Neither equity focused nor unfocused	4= Equity focused	5 = Highly equity focused
○	○	○	○	○

21. Do you think that this is a good example of a highly equity focused quotation? Do you have another example in mind that might be better?

Equity in guideline development handbooks - Domain 5

Quotation 5 - Updating

“Proposals on the need to update a guideline are based on:

- an assessment of the event and any other relevant evidence published since guideline publication (abstracts of primary or secondary evidence)
- information from topic expert engagement,
- if relevant, intelligence gathering and feedback from stakeholder consultation”

22. How equity focused is this quote when considering updating?

1 = Not at all equity focused	2 = Slightly equity focused	3 = Neither equity focused nor unfocused	4= Equity focused	5 = Highly equity focused
○	○	○	○	○

23. Do you think that this is a good example of a highly equity focused quotation? Do you have another example in mind that might be better?

Equity in guideline development handbooks - Domain 5

Quotation 6- Updating

"In cases where new and potentially relevant evidence has been identified, the co-chairs need to assess whether this new evidence warrants a modification of current recommendations. Situations in which an evidence-based guideline necessitates updating include changes in available interventions, in evidence on benefits or harms of available interventions, in new population targeted, in the values placed on important outcomes, in the resources available in healthcare, or if the evidence results in practice changes."

24. How equity focused is this quote when considering updating?

1 = Not at all equity focused	2 = Slightly equity focused	3 = Neither equity focused nor unfocused	4= Equity focused	5 = Highly equity focused
○	○	○	○	○

25. Do you think that this is a good example of a highly equity focused quotation? Do you have another example in mind that might be better?

Equity in guideline development handbooks - Domain 5

Thank you!

Thank you for participating in this survey! Your feedback will help us further develop the tool!

S3 - Instructions for pilot testing the EquAT

S3 - Instructions for pilot testing the EquAT

Pilot testing instructions Using the WHO Handbook for Guideline Development, 2nd edition (2014)

Goal of pilot test:

- Assess how well the "WHO guideline development handbook" considers health equity.
- Provide feedback and suggest any changes to the tool

Instructions:

1. Read the essential element in the equity assessment tool for guideline development handbooks (starts on pg. 10 of the doc "2-Equity_assessment_tool_9.24")

2. Locate the section of the handbook where you can find that essential element. For your ease, I have provided page numbers and sections in the cheat sheet below (pg. 3). Free to read other sections or chapters.

3. Score the guideline development handbook using the equity assessment tool. To score:
    a. Assess how well the sections that you read align with the criteria and considerations.
    b. Criteria are explicit text or material that should be in the content of the guideline development handbook. Additional considerations may also be used in your judgment of scoring.
    c. The more criteria and considerations that the guidelines development handbook section meets, the higher you should score the essential element.

Comments box

Each essential element in the tool has a comments box. Use this box for comments on:

1. The rationale for your score
2. How to improve the clarity of language
3. You can also copy paste any quotes that you think are relevant to why and how you made your assessment

You can also make track changes or add comments in the google doc.

Instructions for scoring essential elements

Score of 0 (the section of the guideline development handbooks meets none of the criteria and considerations of the essential element in the tool). A score of 0 means that none of the content in the guideline development handbook aligns with the criteria and considerations. The section of the guideline development handbook is not equity focused.

Score of 4 (the section of the guideline development handbooks meets all criteria and considerations of the essential element in the tool). A score of 4 means that the content of the guideline development handbook meets all criteria and considerations in the tool. The section of the guideline development handbook is highly equity focused.

Scores between 1-3. Assign scores 1-3 when the content of the guideline development handbook meets some of the criteria or considerations. Scores are assigned depending on the completeness of quality and reporting in the guideline development handbook. The more criteria and consideration met in the guidelines development handbook, the higher you should score the guideline development handbook.

WHO Handbook for Guideline Development 2nd Edition Instructions

Below are sections within the WHO guideline development handbook that will help refer to that essential element. Feel free to read other sections of the handbook. The cheat sheet is just to help you locate elements quickly.

- *Chapters less frequently referenced (please review)*
  - *Chapter 1* – summarizes types of guideline projects and there is more detail in the rest of the book
  - *Chapter 11 Rapid advice guidelines in the setting of a public health emergency* – please look at this entire chapter as it is a mini–guideline development handbook for this topic and covers: scoping, identifying literature, synthesis evidence, developing a report, etc.
  - *Chapter 17* is specific to diagnostic tests and less relevant for this tool
  - *Criteria for use of evidence to inform recommendations* – please look at these pages. They are overarching principles for the authoring organization
- *Chapters highly relevant*
  - *Chapter 5* is on incorporating equity, human rights, gender, and social determinants into guidelines. I reference it down below in certain sections, but it might be useful to look at the entire chapter.

Essential element	Where to find the information
#1 Defining a topic	● Chapter 2 (especially 2.1-2.6)<br>● Chapter 4 (table 4.1)<br>● Chapter 5 (see section 5.4.1.1, 5.4.1.2)<br>● Chapter 5 (box 5.1)<br>● Chapter 15 (section 15.2.1)<br>● Chapter 18 (section 18.3, 18.4.3)
#2 Determining the scope	● Chapter 2 (section 2.7)<br>● Chapter 5 (section 5.4.1.2)<br>● Chapter 5 (box 5.1 #3)<br>● Chapter 15 (section 15.2.1)<br>● Chapter 18 (18.4.2, 18.4.3, also see table 2)
#3 Identifying existing guidelines, adapting, and adopting	● Chapter 1 (see section 1.8.3)<br>● Chapter 2 (section 2.1-especially 2.1.3)<br>● Chapter 5 (see section 5.4.3.2)<br>● Chapter 13 (all)<br>● Chapter 15 (15.2.4)<br>● Chapter 18 (section 18.6)
#4 Forming guideline development group	● Chapter 3 (see section 3.1-3.9)<br>● Chapter 5 (section 5.4.1.3, and box 5.1 #8)<br>● Chapter 16 (especially section 16.12.1)<br>● Chapter 18 (section 18.4.1)

#5 Involving special subgroups	• Chapter 3 (section 3.2.1.3, 3.2.1.4, 3.2.1.5)<br>• Chapter 5 (section 5.4.1.3)<br>• Chapter 15 (section 15.2.2)<br>• Chapter 18 (section 18.4.1)
#6 Managing conflicts of interest	• Chapter 3 (section 3.7)<br>• Chapter 5 (section 5.4.1.4)<br>• Chapter 6 (all)
#7 Establishing clinical questions	• Chapter 5 (section 5.4.1.5)<br>• Chapter 7 (section 7.2-7.4, 7.7)<br>• Chapter 15 (section 15.2.5, 15.3.1)<br>• Chapter 18 (section 18.5.1)
#8 Outcome consideration	• Chapter 5 (section 5.4.1.5)<br>• Chapter 7 (section 7.6-7.7)<br>• Chapter 15 (section15.2.2-15.2.6)
#9 Systematic search for evidence and evidence inclusion	• Chapter 5 (section 5.4.2.1,)<br>• Chapter 8 (8.3 [step 2, step 3, step 4] 8.4, 8.5)<br>• Chapter 15 (section 15.3.2)
#10 Summarizing evidence	• Chapter 5 (section 5.4.2.1,)<br>• Chapter 8 (8.3 [step 5, step 6], 8.4-8.6)<br>• Chapter 15 (section 15.3.3)<br>• Chapter 18 (section 18.5.2)
#11 Appraising evidence	• Chapter 5 (section 5.4.2.2)<br>• Chapter 9 (all)<br>• Chapter 10 (section 10.2.6)<br>• Chapter 14 (particularly section 14.5)<br>• Chapter 15 (section 15.3.4)<br>• Chapter 18 (section 18.5.3)
#12 Conducting economic evaluation	Please skip scoring this essential element. This is not applicable to the WHO guideline development handbook, and should be scored 999.
#13 Creating recommendations	• Chapter 5 (section 5.4.2.3)<br>• Chapter 10 (all)<br>• Chapter 15 (section 15.4, look at examples tables 1 and table 2 in chapter 15)<br>• Chapter 18 (section 18.5.4 and Annex 1)
#14 Wording and considering ethics	• Chapter 5 (section 5.4.3.1)<br>• Chapter 10 (section 10.6)<br>• Chapter 12 (section12.3)
#15 Reporting and consulting experts	• Chapter 2 (section 2.7.1 bullet point "review")<br>• Chapter 12 (section 12.2)

#16 Dissemination and publications	• Chapter 2 (section 2.4.2)<br>• Chapter 5 (section 5.4.3.1)<br>• Chapter 12 (section 12.4)
#17 Implementation and evaluation	• Chapter 5 (section 5.4.3.2)<br>• Chapter 13 (section 13.2-13.3)<br>• Chapter 15 (section 15.2.4)
#18 Updating	• Chapter 12 (12.5)

S4 - Thematic codes applied to feedback from survey data open ended responses

S4-Thematic codes applied to feedback from survey data open ended responses

Expert feedback category*	Definition of feedback category	Example of feedback to which we applied the code**
Addition of language to existing criteria	Respondent describes adding clarifying language to the tool	•"For Criteria 1 can we say any form of Systematic evidence synthesis, I think it can go beyond a scoping review. Under criteria two, can the last words read other experts from diverse backgrounds?" (Essential element #2 Determining the scope)<br><br>•"Instead of allow comment, suggest invite comment" (Essential element #15 Reporting and consulting experts)<br><br>•"Criteria 1 involves some judgment from panel members especially when evidence is lacking or poor quality for population subgroups. This can be subjective. Criteria 3, use of a framework may be more objective. It is not clear if you are suggesting that a guideline developer should use or include all these in their handbook or use one or more." (Essential element #13 Creating recommendations)

Expert feedback category*	Definition of feedback category	Example of feedback to which we applied the code**
Changing terminology related to subgroups	Respondent calls out a certain terminology related to certain subgroups	•"We received feedback that the term 'disadvantaged groups' may not be an accurate description of the cohorts that require consideration in an equitable guideline. For example, war veterans are not necessarily disadvantaged but may require special consideration in mental health guidelines. Further feedback received from minority religious and ethnic groups indicated that they perceive this term unfavorably when it is used to refer to them. You may want to consider 'special needs groups' as a term or seek advice or user test a number of terms with consumers." (Essential element #1 Defining a topic)<br><br>•"I wanted to highlight the colonial roots of the term "Stakeholder" in which a stake was put into land to demarcate land being stolen from Indigenous People. Continued use of this term may be disrespectful and unequitable - suggest you acknowledge this and/or consider another term. " (Essential element #4 Forming guideline development groups"
Adding reference	Respondent describes adding a reference to the criteria or asks that the assessor look up reference	•"…Also, important to consider risk of stigmatizing patient groups when dedicating a part of the guideline to a specific subgroup. See this thought-provoking example of misunderstanding what is root causes (and what is immediate effects) and of causing stigma in guidelines development" (Essential element #1 Defining the topic)

Expert feedback category*	Definition of feedback category	Example of feedback to which we applied the code**
Addition of criteria to essential element	Adding new criteria to the essential element	•"Consider new or emerging literature describing inequities" (Essential element #3 Identifying existing guidelines, adapting, and adopting)<br><br>•Documenting the adaptation/adoption process (Essential element #3 Identifying existing guidelines, adapting, and adopting)
Do not understand	Respondent does not understand the criteria. This might be because there is too much information or because	•"I don't understand clearly this element: there are many aspects included: It is difficult to think in the all aspects included with a punctuation" (Essential element #3 Identifying existing guidelines, adapting, and adopting)
Unclear change to tool	Unclear feedback or feedback that cannot be incorporated in to the tool	•"While these criteria are clearly relevant, and ensuring equity, diversity and inclusion in the guideline group is crucial to addressing equity well, the criteria are quite high level and don't really provide the practical guidance about how to think through these issues - or tools to help with that like the Progress+ criteria, etc. So I'd rate them reasonably high on relevance, I wouldn't rate them very high on helpfulness." (Essential element #1 Defining a topic)
No feedback	No additional feedback from the respondent, or the field is left blank	•"None"
Adding to another part of the tool	Respondent suggests additional language but that change should be applied to the other parts of the item, like title or definition. It could also apply when changes are for the instructions of the tool	•"We would also support a representative on the panel if there are likely to be substantial impacts for specific populations in addition to topic prioritization." (Essential element #1 Defining a topic)

Expert feedback category*	Definition of feedback category	Example of feedback to which we applied the code**
Changing the order of criteria or considerations in the tool	Respondent suggests that we change the order of the criteria to emphasize different parts of the criteria	• "I would prioritize point 2 over point 1 and probably clarify that point 3 is part of the substantive literature review process rather than at the scoping stage. (Essential element #2 Determining the scope)"

*Criteria are explicit equity-focused language that an assessor should look for in a guideline development handbook

**Two open ended questions were "Do you have feedback on additional criteria" and "Do you have suggestions to improve the clarity of these criteria"

S5 - Ratings of experts on equity focused quotations from guideline development handbooks

S5 - Ratings of experts on equity focused quotations from guideline development handbooks

We presented experts with two quotations from various guideline development handbooks for each of the "essential elements." We asked experts to rate equity content on a scale of 1-5 (1=not at all equity focused, 5= highly equity focused). We blinded experts to the source of the quotations.

Domain 1 – Preparing for the guideline

Essential element #1 Defining a topic		
**Quotation**	**Guideline Development Handbook**	**Ratings**
"The planned achievements should focus not only on the average level of health, but also how health is distributed within populations and across groups. The idea is to ensure that those of lower social position and with greater need can benefit more than advantaged persons"	WHO	Expert 1 = 4<br>Expert 2 = 2<br>Expert 3 =4<br>Expert 4 =2<br>Expert 5 =4<br>Expert 6 =4
"Considerations must include the link between the topic and national health-care priorities, and/or the relevance of the guideline.<br><br>6.2.1.1 *Burden of disease in Estonia*- It is important to consider the size of the patient/target group(s) affected by the disease or condition in Estonia (morbidity, prevalence, mortality, etc.).- The impact of the disease or condition on the Estonian health and social care system should also be taken into account.<br>6.2.1.2 *Differences in practice and/or health outcomes and/or costs*- Significant differences exist in practices and between/within patient groups (including subgroups); by health-care providers and/or different levels of care (e.g. primary care versus specialist medical care); in different regions of Estonia; or by different cost categories (medicinal products, inpatient treatment, etc.).- Differences also exist between Estonian and international practices.<br><br>6.2.1.3 *Expected impacts on patient health indicators and/or use of resources*-Anticipated impacts include:- modernizing current practices;- introduction of new interventions (including diagnostic and other tests and health-care services);- availability of new evidence-based practices, possibly altering current practices;- more efficient use of resources."	Estonia national handbook	Expert 1 = 5<br>Expert 2 =1<br>Expert 3 =4<br>Expert 4 =1<br>Expert 5 = skipped<br>Expert 6 =3

Essential element #2 Determining the scope		
**Quotation**	**Guideline Development Handbook**	**Ratings**
"During the development of the scope, it is important to consider and assess any health equality and equity issues to establish:<br>• whether there is any risk of unlawful discrimination arising from the guideline<br>• whether the guideline offers any opportunity for advancing equality or reducing inequalities<br>• whether there might need to be reasonable adjustments to a recommendation to avoid putting any group of people covered by the scope at substantial disadvantage<br>• whether and to what extend particular equality issues should be included in the scope"	NICE	Expert 1 = 5<br>Expert 2 =4<br>Expert 3 =5<br>Expert 4 =4<br>Expert 5 =5<br>Expert 6 =4
"**Determine Guideline Scope**<br><br>**Affected Population**: Developers might categorize affected populations by age (e.g., newborn babies), behavior (e.g., men who have sex with men), geography (e.g., populations in states in the Pacific Northwest), occupation (e.g., health care workers) or other criteria.<br><br>**Intended Audience**: Public health guidelines often have diverse audiences (e.g., practitioners, policy makers, health care businesses, and government agencies); therefore, they will need to balance the information according to multiple needs.<br><br>**Guideline Setting:** Settings might include doctors or dental offices, hospitals, nursing homes, day care facilities, schools, colleges, workplaces, pharmacies, supermarkets, or the community at large. Settings might be narrowed by the populations they serve. For example, school-based guidelines might focus on schools in low-income areas, which could be determined by factors such as the proportion of children who are eligible for free school lunches"	CDC 2022	Expert 1 = 5<br>Expert 2 =4<br>Expert 3 =4<br>Expert 4 =4<br>Expert 5 =5<br>Expert 6 =2

Essential element #3 Identifying existing guidelines, adapting and adopting		
**Quotation**	**Guideline Development Handbook**	**Ratings**
"**Adopting or adapting a guideline**: An 'equity lens' is a series of structured questions focusing on the visibility of equity, inequity and socioeconomic determinants, can be used to assess whether existing guidelines have explicitly considered the needs and values of disadvantaged populations."	NHMRC equity section	Expert 1 = 4<br>Expert 2 = 4<br>Expert 3 =5<br>Expert 4 =2<br>Expert 5 =5<br>Expert 6 =5
"5.4.3.2 Adaptation, implementation and evaluation (Chapter 13)<br><br>When developing guidelines, clear references to health equity, the social determinants of health, gender equality and relevant international human rights standards and principles, should be included in the sections on implementation, monitoring and evaluation. The interventions recommended in a guideline pose the risk of increasing rather than reducing inequities, depending on how they are implemented. For example, marginalized population groups might be ignored or strategies that make them the last to benefit might be used. Important gender differences might not be considered or unintended effects on certain subpopulations or population strata might occur. Such situations arise because decisions governing implementation are not explicitly related to equity. For instance, vaccination coverage targets might be achieved without benefit to the poorest and most disadvantaged groups. WHO staff have a responsibility to monitor and evaluate how recommendations affect equity, human rights, gender equality and the advancement of the highest attainable standard of health, and how health services and programmes implement the recommended interventions."	WHO	Expert 1 = 4<br>Expert 2 =5<br>Expert 3 =5<br>Expert 4 =2<br>Expert 5 =5<br>Expert 6 =5

Domain 2 – Identifying the guideline group

Essential element #4 Forming guideline development group		
**Quotation**	**Guideline Development Handbook**	**Ratings**
"Group membership should not only reflect diversity, including geographical, gender balance or cultural background, but should also include populations with where the health conditions are more prevalent…all guidelines groups should try to include at least one indigenous representative…."	(NHMRC, Guideline group development "Determine the Structure and Complexity of the Group," online)	Expert 1 = 4<br>Expert 2 =4<br>Expert 3 = 4<br>Expert 4 = 5
"The Panel must represent a balance of the various health-care levels (primary care, hospital care, nursing care) according to the topic. It monitors the regional representation of experts and involves health professionals whose work will be most affected by the guideline. Panel members represent their own views and not those of organizations, although they may be recruited from or suggested by these organizations. The optimal Panel size is 8–10 members, but depending on the guideline topic and target group, it may be necessary to involve fewer or more members and to invite consultants for their input on individual issues."	Estonia	Expert 1 = 4<br>Expert 2 = 1<br>Expert 3 = 1<br>Expert 4= 5

Essential element #5 Involving special subgroups		
**Quotation**	**Guideline Development Handbook**	**Ratings**
"Some approaches to consider include:<br><br>• formal, structured activities (e.g., interviews, focus groups, consensus methods)<br>• informal methods (e.g., project or advisory meetings, feedback on documents, public consultations<br>• modes of involvement (e.g., face-to-face, online, video-/teleconference, email)<br>• timing of involvement (e.g., one-ff, multistage, continuous)<br><br>You will need to be flexible and adapt to consumer needs for any approach you choose to take, especially if they live remotely or have ongoing health challenges"	NHMRC consumer involvement	Expert 1 = 5<br>Expert 2 = 2<br>Expert 3 = 1<br>Expert 4 = 4
"Stakeholder engagement in guideline development enhances the relevance of the guidelines, creates a sense of ownership among stakeholders, and raises awareness about the project, ultimately facilitating acceptance, implementation, and adherence. It also contributes to the accountability and legitimacy of the guideline developers. Stakeholder engagement requires careful planning in terms of whom to engage, at which steps of the guideline development process, at what level of engagement, and using which mode. The following sections address these four questions. In addition, practical guidance on facilitators for stakeholder engagement, reporting on stakeholder engagement, and evaluation of stakeholder engagement are referenced."	KDIGO Guideline development handbook	Expert 1 = 5<br>Expert 2 = 1<br>Expert 3 = 1<br>Expert 4 = 4

Essential element #6 Managing conflict of interest		
**Quotation**	**Guideline Development Handbook**	**Ratings**
"Can the interest consistently produce bias?<br><br>Consider whether the interest would produce a consistent direction of bias in the context of guideline development, such as if financial sponsorship were to influence decisions consistently in favor of an intervention or product preferred by the sponsor"	NHMRC guidebook	Expert 1 = 5<br>Expert 2 = 2<br>Expert 3 = 1<br>Expert 4 = 3
Types of COI:<br><br>"Intellectual or academic - occurs when a person or a professional group is jeopardized or enhanced by a guideline recommendation. Guyatt defined this as ''academic activities that create the potential for an attachment to a specific point of view that could unduly affect an individual's judgment about a specific recommendation' [Guyatt G et al. 2010]. Examples include having published a scientific paper on the topic, having received grant support related to the guideline, having personal beliefs related to the topic that may lead to biased writing and publishing, being a chair or member of another guideline committee relevant to the topic, involvement in an advocacy group that stands to benefit from recommendations, being a member of a lobbying or advocacy organization related to the topic, or having family members with the condition addressed by the CPG."	Philippines national guidebook	Expert 1 = 4<br>Expert 2 = 1<br>Expert 3 = 1<br>Expert 4 = 1

Domain 3 - Gathering the evidence

Essential element #7 Establishing clinical questions		
**Quotation**	**Guideline Development Handbook**	**Ratings**
"For each review question, factors that may affect the outcomes and effectiveness of an intervention, including any wider social factors that may affect health and any health inequalities, should be considered"	NICE	Expert 1 =2<br>Expert 2 =3<br>Expert 3 =4<br>Expert 4 =4

Essential element #8 Outcome consideration		
**Quotation**	**Guideline Development Handbook**	**Ratings**
"**Patient important outcomes** should be explicitly considered along with more narrowly defined clinically important outcomes. It is particularly important to include any potential harm associated with the intervention under review so that a balanced view can be taken at the considered judgement stage."	*SIGN 50 guidebook, pg. 16*	Expert 1 =4<br>Expert 2 =3<br>Expert 3 =4<br>Expert 4 =3

Essential element #9 Systematic search for evidence and inclusion		
**Quotation**	**Guideline Development Handbook**	**Ratings**
"Databases relevant to these groups should be identified and searched, such as the Informit Indigenous Collection and the Aboriginal and Torres Strait islander Health Bibliography (Aboriginal and Torres Strait Islander Health, compiled by the Australian Indigenous Health Infonet), both of which provide searchable databases of literature about Aboriginal and Torres Strait Islander health"	*(NHMRC, "Equity," "What to do," online)*	Expert 1 =4<br>Expert 2 =4<br>Expert 3 =5<br>Expert 4 =4

Essential element #10 Summarizing the evidence		
**Quotation**	**Guideline Development Handbook**	**Ratings**
"One of the key objectives for evidence synthesis is to explore the reasons for difference in observed effects and to identify any populations or interventions/exposure categories that are associated with these differences. This can be a critical area of investigation used to inform a guideline's recommendations to support specific actions, for different populations. It is especially relevant to considerations of equity."	*(NHMRC, "Synthesizing evidence" "Investigate the reasons for different effect")*	Expert 1 =5<br>Expert 2 =4<br>Expert 3 =4<br>Expert 4 =4

Essential element #11 Appraising the evidence		
**Quotation**	**Guideline Development Handbook**	**Ratings**
"The concept of indirectness refers to whether the evidence available, including the population, comparisons and outcomes measured, directly and completely answers the question proposed by the guideline…examples of important indirectness might include:<br><br>• Randomized trials of narrow segments of the population such as only participants with relatively mind illnesses or only adult,<br>• Studies conducted in high-income, urban settings rather than including rural or low-income settings…"	• *(NHMRC, Assessing certainty of evidence, online)*	Expert 1 =2<br>Expert 2 =4<br>Expert 3 =5<br>Expert 4 =4

Essential element #12 Economic evaluation		
**Quotation**	**Guideline Development Handbook**	**Ratings**
"to support our commitment to addressing health inequalities, we have commissioned a prototype tool to explore the approach of providing quantitative estimates of the impact of NICE recommendations on health inequalities. The tool uses distributional cost-effectiveness analysis to model changes in health inequalities between 5 socioeconomic groups in England based on the neighborhood index of multiple deprivation"	NICE pg. 188	Expert 1 =4<br>Expert 2 =4<br>Expert 3 =4<br>Expert 4 =4

Domain 4- Drafting and review

Essential element #13 Creating recommendations		
**Quotation**	**Guideline Development Handbook**	**Ratings**
"The committee should also assess the extent to which recommendations may impact on health inequalities. This needs to be made clear, regardless of whether the recommendation is aimed at the whole population, specific subgroups or a combination of both")	(NICE, pg. 206	Expert 1= 5<br>Expert 2= 4<br>Expert 3 = 4
"5.4.2.3 Developing recommendations (Chapter 10)<br><br>The effect of an intervention on equity is one of the factors that determine the strength of a recommendation: if an intervention is likely to reduce health inequities, a strong recommendation may be warranted, provided it is justifiable in light of other factors. On the other hand, if equity is a key consideration and evidence on the intervention's potential effects on equity is sparse, a conditional recommendation may be appropriate. In the latter case particularly, the guideline should outline the key gaps in knowledge and provide an agenda for future research. Evaluation and monitoring of the impact of recommendations that potentially affect inequities are also critically important and should be articulated in the guideline document."	WHO	Expert 1= 5<br>Expert 2=5<br>Expert 3 = 5

Essential element #14 Wording and ethics		
**Quotation**	**Guideline Development Handbook**	**Ratings**
"Avoid labelling people. Conditions describe what the person has, not what the person is. Diseases are treated, not people. Diseases, not people, respond to treatment. Conditions, not people, are monitored. People are not unsuitable for treatments: treatments are unsuitable for them. People have diseases, they do not suffer from them"	*From NICE style guide "talking about people"*	Expert 1= 5<br>Expert 2=3<br>Expert 3 = 1
"Throughout the process careful attention should be paid to the use of language, the selection of case examples, the expressions used to refer to population groups, the choice of photographs, etc. Setting the ground rules up front is much easier than trying to modify a document that is ready for layout. In translations of guidelines, gender-sensitive and non-stigmatizing language should always be employed."	WHO	Expert 1= 4<br>Expert 2=4<br>Expert 3 = 5

Essential element #15 Reporting and consulting experts		
**Quotation**	**Guideline Development Handbook**	**Ratings**
"Developers must take the following key points into account when responding to comments from registered stakeholders:<br><br>• Each comment must be acknowledged and answered as directly, full and with as much information as possible…<br>• For a draft guideline, the committee must consider whether changes to the guideline are needed as a result of consultation comments; any changes to the guideline must be agreed by the committee before publication.<br>• If changes are made to a guideline as a result of a consultation comment, this must be made clear in the response to the comment. If no changes have been made, it should be clear from the response why not..."	NICE	Expert 1= 3<br>Expert 2=3<br>Expert 3 = 1
"Public consultation methods can be either open, targeted or a combination of the two (GIN Public 2012). By inviting specific stakeholders to comment, targeted consultation methods enable feedback to be sought in a relatively controlled manner; however, this also runs the risk of important viewpoints being overlooked. Open consultation is more transparent and can capture views from individuals or groups that might not otherwise have been planning or expected to engage."	NHMRC	Expert 1= 4<br>Expert 2=4<br>Expert 3= 2

Domain 5 – Dissemination, implementation, evaluation, and updating

Essential element #16 Dissemination and publications		
**Quotation**	**Guideline Development Handbook**	**Ratings**
"NICE may use a range of different methods to raise awareness of the guideline. These include standard approaches such as:<br><br>• notifying registered stakeholders of publication<br>• publicizing the guideline through NICE's newsletters and alerts<br>• issuing press release as appropriate, posting news articles and blogs on the NICE website, using social media channels and promoting the guideline within NICE"	NICE	Expert 1 = 1<br>Expert 2 = 4<br>Expert 3 = 4
"Patients as champions for change<br><br>Patients are a powerful agent for change in the health service. Guidelines are published with an accompanying patient and carer version of the guideline and by being aware of a clinical guideline, patients can ask for their care to be in line with the latest recommendations. Making use of connections with patient groups and voluntary organisations also affords more opportunities to raise awareness of guidelines. Lay representatives on guideline development groups are supported to raise awareness at conferences and other events."	SIGN 50	Expert 1 = 4<br>Expert 2 = 4<br>Expert 3 = 2

Essential element #17 Implementation and evaluation		
**Quotation**	**Guideline Development Handbook**	**Ratings**
"Topic experts (including members of NICE's Expert Advisers Panel) are invited to participate in surveillance. They provide their views about how an event affects the recommendations, and their knowledge of recent developments in the topic area. If the response from topic experts is limited, or further specialist input is needed, we may seek input from other experts."	NICE	Expert 1 = 1<br>Expert 2 = 3<br>Expert 3 = 2
"2. Gather information about implementation throughout development<br><br>Discussions about guideline implementation may happen in the initial scoping phase of a guideline when evidence-practice or knowledge gaps are being identified. If a guideline already exists in this field, but is presumably out of date, the first question you should ask is whether it was implemented and, if so, whether it was implemented successfully. Evaluation data are rarely available for guidelines in Australia (NHMRC 2014) and so it is likely that you will need to gather this information through stakeholder consultation. In this initial phase of guideline development it is important to document any discussions or feedback, whether formal or informal, regarding implementation ideas to inform future activities."	NHMRC	Expert 1 = 1<br>Expert 2 = 5<br>Expert 3 =

Essential element #18 Updating		
**Quotation**	**Guideline Development Handbook**	**Ratings**
"Proposals on the need to update a guideline are based on:<br><br>• an assessment of the event and any other relevant evidence published since guideline publication (abstracts of primary or secondary evidence)<br>• information from topic expert engagement,<br>• if relevant, intelligence gathering and feedback from stakeholder consultation"	NICE	Expert 1 = 2<br>Expert 2 = 3<br>Expert 3 = 2
"In cases where new and potentially relevant evidence has been identified, the co-chairs need to assess whether this new evidence warrants a modification of current recommendations. Situations in which an evidence-based guideline necessitates updating include changes in available interventions, in evidence on benefits or harms of available interventions, in new population targeted, in the values placed on important outcomes, in the resources available in healthcare, or if the evidence results in practice changes."	IDSA guidebook	Expert 1 = 2<br>Expert 2 = 4<br>Expert 3 = 4

S6- Reactions of four reviewers to an example of an equity focused quotation from a guideline development handbook

S6- Reactions of four reviewers to an example of an equity focused quotation from a guideline development handbook

<table>
<tr><td colspan="3">Example quote shown to reviewers for essential element #4 Forming guideline development group:

“The Panel must represent a balance of the various health-care levels (primary care, hospital care, nursing care) according to the topic. It monitors the regional representation of experts and involves health professionals whose work will be most affected by the guideline. Panel members represent their own views and not those of organizations, although they may be recruited from or suggested by these organizations. The optimal Panel size is 8–10 members, but depending on the guideline topic and target group, it may be necessary to involve fewer or more members and to invite consultants for their input on individual issues.”*</td></tr>
<tr><th>Expert</th><th>Score**</th><th>Comment</th></tr>
<tr><td>#1</td><td>4</td><td>"Overall I like this quote. My only issue is with the word "levels" ("health-care levels"). Primary care, hospital care, and nursing care are not really 3 different "levels"- the word implies a hierarchy. They are just 3 different specialties or disciplines. I also wonder about the last sentence, "it may be necessary to involve fewer members"-- more members is OK, but fewer members suggests that this could be used as a loophole or an "out" in a challenging situation.</td></tr>
<tr><td>#2</td><td>1</td><td>"Nothing in this statement is particularly equity-focused to me - this just seems like standard practical advice. To be equity focused, you could consider adding "...various health care levels (primary care...) and resource settings (low-vs high income, underserved vs well-resourced, urban/rural). I also disagree that "panel members represent their own views" when sometimes/often a particular panel member is recruited because they are a representative of a larger group. E.g., being a patient group advocate. Do you have any recommendations for a % of the panel that should be represented by patients or equity-deserving groups?</td></tr>
<tr><td>#3</td><td>1</td><td>"No feedback"</td></tr>
<tr><td>#4</td><td>4</td><td>"This is equity focused in some ways, but not in other ways"</td></tr>
</table>

*This quote comes from the “Estonian Handbook For Guidelines Development" (2020), however, survey respondents were blinded to the source

** Scale for this question is: 1=Not at all equity focused, 2=Slightly equity focused, 3= Neither equity focused nor unfocused, 4= Equity focused, 5= Highly equity focused

S7- Final EquAT tool

Equity Assessment Tool for Guideline Development Handbooks
(EquAT tool)

User Guide and Tool

November, 2025

Table of contents

Background 3

Objective and intended audience 3

Rationale 3

Application 4

Tool instructions 4

Preparing for assessment 4

Development and validation of this tool 5

Essential elements and domains 5

Item structure for each "essential element" 6

Conducting the assessment for each "essential element" 6

Analysis of scores 8

Domain 1 - Planning for the guideline 9

Essential element #1 Defining a topic 10

Essential element #2 Determining the scope 11

Essential element #3 Identifying existing guidelines, adapting, and adopting 13

Domain 2 − Identifying the guideline group 15

Essential element #4 Forming guideline development group 16

Essential element #5 Involving special subgroups 18

Essential element #6 Managing conflicts of interest 20

Domain 3 − Gathering the evidence 21

Essential element #7 Establishing clinical questions 22

Essential element #8 Outcomes Consideration 23

Essential element #9 Systematic search for evidence and evidence inclusion 24

Essential element #10 Summarizing the evidence 25

Essential element #11 Appraising the evidence 26

Essential element #12 Conducting economic evaluation 27

Domain 4 − Drafting and review 28

Essential element #13 Creating recommendations 29

Essential element #14 Wording and considering ethics 31

Essential element #15 Reporting and consulting experts 32
Domain 5 − Dissemination, implementation, evaluation, and updating 33
Essential element #16 Dissemination and publications 34
Essential element #17 Implementation and evaluation 35
Essential element #18 Updating 36
References 37

Background

Objective and intended audience

The objective of The Equity Assessment Tool for Guideline Development Handbooks (EquAT) tool is to foster discussions on health equity considerations within guideline development handbooks. The EquAT tool is intended for both authors of guideline development handbooks and groups determining which work they wish to use as a part of their processes. Guideline development handbook authors may use this tool to 1) audit their own guideline development handbook against health equity practices proposed in this tool and 2) foster discussions amongst themselves on ways to enhance health equity considerations in their guideline development process.

Rationale

Clinical practice guidelines (CPGs), as defined by the Institute of Medicine (IOM now the National Academies) in their 2011 publication, are “statements that include recommendations intended to optimize patient care that are informed by systematic review of evidence and an assessment of benefits and harms of alternative care options” (1). Since the 1990s, CPG publication has rapidly accelerated. With the increase in guideline production, organizations developed methods to improve the quality and consistency of evidence-based guidelines. **Guideline development handbooks** provide guideline development panels with instructions on how to create clinical practice/public health guidelines (like defining a topic, generating review questions, developing a search strategy, and drafting recommendations, etc.). Professional organizations for clinical conditions, health services researchers, and governmental departments refer to guideline development handbooks during their processes (2). These works may also promote ideas and best practices to other guideline development groups (3).

A commonly cited definition for health equity is proposed by Whitehead et al who note that inequities are “differences which are unnecessary and avoidable, but in addition, are also considered unfair and unjust ”(4). Braveman and Gruskin adds to this definition noting that health equity is the same as social justice, an ethical concept about the fair distribution of health (5). Economists agree and note that health equity is both a moral and practical endeavor. Health is central to human life and capability, and as such, good health affords people opportunities (6, 7). Clinical practice guidelines are one way to addresses health inequities, and make healthcare “more fair”(8). Ignoring health equity in clinical practice/public health guidelines, and the methods that create them, may result in misdiagnosis, poor access to health, and widening of health inequities for groups at higher social risk (9, 10).

Earlier works on health equity and guideline development are informative and offer extensive guidance on when and how to consider health equity during the process. (11-18). However,

organizations still rely on guideline development handbooks for guidance. This tool focuses on the extent to which guideline development handbooks support health equity.

Application

Audiences may apply the EquAT tool to any international or national evidence-based guideline development handbook that includes CPG and/or public health guidelines. This tool **does not apply** to the following:

1. Clinical practice or public health guidelines that stand alone (i.e. not in a handbook)
2. Good/best practice statements that stand alone
3. Opinion based, consensus or non-evidence-based guidance development handbooks (2)
4. Processes for generating good/best practice statements.
5. Protocols, statements, technical reports, conference notes that may provide guidance for guidelines development.
6. Appropriate use criteria guidance handbooks or documents (AUC)
7. Standard treatment guidelines (STG) development handbooks (since these handbooks are often adapted from international guidelines development handbooks and might include a list of essential medication for a population)

These seven types of works are either developed outside the context of a guideline development handbook (#1, #2, #5) or are distinct processes compared to from evidence based clinical practice guideline development (#3, #4, #6, #7).

Tool instructions

Preparing for assessment

Before using the tool, assessors should familiarize themselves with the structure of the guideline development handbook. Assessors should review the table of contents, appendices, and supplemental information. Guideline development handbooks vary in their presentation. Some guideline development handbooks are available only online and require click-through, while others are pdf documents. Identifying parts of the guideline development handbook that are relevant to items in the tool beforehand will help assessor as they move through the tool.

In this tool, there is no specific number of assessors for a given guideline development handbook. During our validation process, we found that assessors might interpret the same text in different ways, leading to a diversity of scoring. As such, we present a series of guiding questions for assessors to discuss. Conversations among these assessors about their reasoning may be valuable in identifying areas of improvement in the guideline development handbook they are evaluating.

Before applying the tool, assessors should consider and discuss:

- Meanings of health equity for their guideline authoring organization/group
- Experience and areas of research or practice emphasis for each assessor
- Any essential elements that the group does not want to assess (applying the 999 score)

Development and validation of this tool

The details of the development and validation of this tool are in the accompanying publication.

Essential elements and domains

The EquAT contains 18 items within 5 domains. Each item in the tool corresponds with an "essential element." Essential elements are tasks that scholars agree are an integral part of guideline creation.

Table 1: List of 18 essential elements under five domains of guideline development identified by comparing three previous publications (Turner et al 2008, Ansari et al 2012, and Schünemann et al, 2014)

Domain	Essential element number and description
Domain 1 – Preparing for the guideline	• #1 Defining a topic<br>• #2 Determining the scope<br>• #3 Identifying existing guidelines, adapting and adopting
Domain 2 – Identifying the guideline group	• #4 Forming guideline development group<br>• #5 Involving special subgroups<br>• #6 Managing conflicts of interest
Domain 3– Gathering the evidence	• #7 Establishing clinical questions<br>• #8 Outcomes consideration<br>• #9 Systematic search for evidence and evidence inclusion<br>• #10 Summarizing the evidence<br>• #11 Appraising the evidence<br>• #12 Conducting economic evaluation
Domain 4– Drafting and review	• #13 Creating recommendations<br>• #14 Wording and considering ethics<br>• #15 Reporting and consulting experts

Domain 5– Dissemination, implementation, evaluation and updating	• #16 Dissemination and publications<br>• #17 Implementation and evaluation<br>• #18 Updating

References: (2, 19, 20)

Item structure for each "essential element"

The structure of the EquAT and the instructions for use are adapted from the previously validated AGREE II tool (21, 22).

Each of the 18 essential element items in the EquAT tool has **six parts**[1]**:**

1. **Essential element** –Name of the "essential element."<br><br>2. **Definition of the essential element -** Provides a brief description of the "essential element."
3. **How to rate a guideline development handbook**– Identifies information the assessor should look for to rate the "essential element."<br><br>a. **Criteria**: Details of explicit language or subject matter an assessor should seek when assessing the handbook.<br><br>b. **Additional considerations**: Details of additional information to consider when rating the guidelines development handbook.
4. **Where to look** – Provides guidance on where the assessor should look for information on the "essential element." This section includes commonly used terms to refer to the "essential element" within guideline development handbooks (21).
5. **Illustrative quote –** Provides an example quotation that fit the criteria for each essential element. These quotations come from guideline development handbooks and are illustrative of potential exemplary language.

6. **Abbreviations**

Conducting the assessment for each "essential element"

To conduct an assessment:

1. Read all parts of the essential element (essential elements 1-18)
2. Locate the section of the guideline development handbook that corresponds to the essential element including any appendices and supplemental information.

[1] Sections for "how to rate," "criteria" "additional considerations" and "where to look" are also adapted from the AGREE II tool (Brouwers et al, 2010)

3. Conduct the assessment (details below)
4. Add any comments in the comments box, including page or section numbers for relevant text, or any justification for the assigned score.

Assessment scale
Assessors may use a 5-point Likert scale to score each essential element:

**0**<br>**Meets none of the criteria NOR considerations**	**1**	**2**<br>**Meets some criteria AND considerations**	**3**	**4**<br>**Meets all criteria AND considerations**	**999**<br>**This essential element does not exist in the guideline development handbook**

Comments:

Score of 0 (Meets none of the criteria nor considerations). A score of 0 means that none of the content in the guideline development handbook aligns with the criteria and considerations. The section of the guideline development handbook is not at all health equity focused.

Score of 4 (**Meets all criteria and considerations**) A score of 4 means that the content of the guideline development handbook meets all criteria and considerations. The section of the guideline development handbook is very health equity focused.

Scores between 1-3. Assign scores 1-3 when the content of the guideline development handbook meets some of the criteria and/or considerations. Scores are assigned depending on the quality and completeness of reporting in the guideline development handbook (21). The more criteria and considerations are met in the guidelines development handbook, the higher an assessor should score the guideline development handbook.

Assign 999 If an “essential element” does not exist in the guidelines development handbook, the assessor may score the item 999. This score is useful for some essential elements that are not always a part of a guideline development process (for instance, not all guideline authoring organizations conduct economic evaluation (essential element #12)).

Comments box Assessors may use the comment box to justify scores. Assessors might include section numbers, or quotes from the guideline development handbook that correspond with their score. When several assessors review a guideline development handbook, the comments box may be used to compare reasoning and text.

Analysis of scores

Scoring for this tool might be subjective. However, we recognize that numerical values are less important than the discussions among assessors for their ratings. Assessors may analyze their results in several ways. They may compare ratings for each individual essential element, and discuss their differences. They may also note their selected passages/sections. They may also calculate domain scores to identify weaker areas in the guideline development handbook.

Optional domain scores (section and formula adapted from AGREE II) (22, 23)

We adapt domain scores as an optional way to further identify areas of improvement for the guideline development handbook, and foster conversations.

- Maximum possible score = (highest score) x (number of items) x (number of assessors) *
- Minimum possible score = (lowest score) x (number of items) x (number of assessors) *
- Obtained score = addition of all the scores within that domain

*If an essential element is scored "999" it is not accounted for in the domain score. This would change "number of items."

The EquAT tool has 5 domains and 18 items. Therefore, use domain scores along with reviewing each essential element individually. Assessors may use domain scores as a place to start seeing patterns of and areas of improvement within their guideline development handbook.

Domain score formula:

$$\frac{Obtained\ score - Minimum\ possible\ score}{Maximum\ possible\ score - Minimum\ possible\ score} \times 100$$

Domain 1 - Planning for the guideline

Incorporate equity considerations into:

- Essential element #1 = Defining a topic
- Essential element #2 = Determining the scope
- Essential element #3=Identifying existing guidelines, adapting and adopting

Essential element #1 Defining a topic

Essential element definition: Selecting guideline topic and defining potential users (19, 20)

0<br>Meets none of the criteria NOR considerations	1	2<br>Meets some criteria AND considerations	3	4<br>Meets all criteria AND considerations	999<br>Essential element not in the guideline development handbook

Comments:

How to rate the handbook– look for discussion of:

Criteria:

1. Prioritizing topics relevant to special subgroups when guideline developers suspect that the distribution of health varies across a population (24, 25).
    a. If a known subgroup requires special attention, consider dedicating a portion of the guideline or creating separate guideline to address the needs of that special subgroup (11). The guideline development handbook may ask guideline panels to acknowledge the potential risk of stigmatizing special subgroups by doing this and request panels provide a justification for a separate or sub guideline.
2. Identifying special subgroups potentially impacted by the recommendations as the target guideline audience (e.g., for example, using any of the PROGRESS-PLUS mnemonic to identify these subgroups) (26).

Considerations:

1. Are the equity criteria easy to find?
2. Are the equity criteria clearly worded?

Where to look

Topic selection is part of the guideline planning process and often appears in the first few chapters. Topic selection might be in the same chapter or section as scoping the guideline. Examine chapters on equity and additional appendices.

Illustrative quote

"The planned achievements should focus not only on the average level of health, but also on how health is distributed within populations and across groups. The idea is to ensure that those of lower social position and with greater needs can benefit more than advantaged persons" *(WHO, chapter 5, "Equity, human rights, gender and social determinants")(27)*

Abbreviations: PROGRESS-PLUS = Place of residence, Race/ethnicity/culture/language, Occupation, Gender/sex, Religion, Education, Socioeconomic Status, Social capital, personal characteristics associated with discrimination (e.g. disability), features of relationships (smoking parents, excluded from school), time dependent relationships

Essential element #2 Determining the scope

Essential element definition: Developers search literature to create a framework that describes the epidemiology of the disease or condition and **"**aspects of care and the setting [that] is covered by the guidelines" (19)

0<br>Meets none of the criteria NOR considerations	1	2<br>Meets some criteria AND considerations	3	4<br>Meets all criteria AND considerations	999<br>Essential element not in the guideline development handbook

Comments

How to rate the handbook – look for discussion of:
Criteria

1. Requiring input on guideline scope from 1) members of potentially impacted special subgroups or 2) other experts from diverse background (11). The guideline development handbook may acknowledge barriers of engagement for special subgroups who cannot contribute to the development process.
2. Any form of systematic evidence review which includes:
   a. Determining the importance or non-importance of equity to guideline development (28).
   b. If the distribution of health effects varies within a population, identifying impacted subgroups as the target audience (11).
   c. Investigating harmful impacts of an action/intervention on special subgroups (11, 29)
   d. See Dans et al's equity lens criteria table 1 for "five criteria to address inequities in guidelines" (30).
3. Substantively reviewing literature with a lens of distribution of health, not just average health effects, to identify the scope of the topic (14). Review of literature may include grey literature or qualitative data to identify epidemiological trends not immediately apparent in other empirical studies.
   a. Consider if reviewed literature provides complete information on equity

Additional considerations

1. Are the equity criteria easy to find?
2. Are the equity criteria clearly worded?

Where to look
Scoping the guideline is part of the guideline planning process and often appears in the first few chapters. Examine chapters on equity and additional appendices.

Illustrative quote
"During the development of the scope, it is important to consider and assess any health equality and equity issues to establish: whether there is any risk of unlawful discrimination arising from the

guideline, whether the guideline offers any opportunity for advancing equality or reducing inequalities, whether there might need to be reasonable adjustments to a recommendation to avoid putting any group of people covered by the scope at substantial disadvantage, whether and to what extend particular equality issues should be included in the scope" *(NICE, chapter 2 "Equity and Equality issues at the scoping stage") (29)*

Essential element #3 Identifying existing guidelines, adapting, and adopting

Essential element definition: Developers search for previous guidelines on the same topic in relevant databases to ensure they are not duplicating work. Guideline development handbooks describe adaptation/adoption methods from other organizations to apply to own population.

0<br>Meets none of the criteria NOR considerations	1	2<br>Meets some criteria AND considerations	3	4<br>Meets all criteria AND considerations	999<br>Essential element not in the guideline development handbook

Comments:

How to rate– look for discussion of:

Criteria

1. Searching previously published guidelines on the same topic to avoid duplication (28). This includes searching for previous guidelines to see how closely they match the current PICO/guideline questions and if these guidelines have an equity lens.
2. Considering if previously published guidelines accounted for equity factors (e.g., if previous guideline content included distribution of health, variation in health services, and outcomes, and identifying target subgroups) (25, 29)
3. Considering new and emerging literature on inequities for specific subgroups (25)
4. When adapting/adopting a guideline:
    a. Considering local context, needs, and differences with the original guidelines (31, 32). Context means "any feature of the circumstance in which an intervention is conceived, developed, implemented and evaluated" (33)
    b. Selecting an appropriate guideline panel and/or advocating for co-creation of guideline (32, 34). This includes a description of selecting primary (representative of potentially impacted subgroups and healthcare workers) and secondary co-creators of guidelines (e.g. healthcare managers, policy makers, NGOs, etc.).
    c. Offering methodological training for guideline developers during adaptation/adoption phase(34, 35)
    d. Documenting the adoption/adaptation process

Additional considerations

1. Are the equity criteria easy to find?
2. Are the equity criteria clearly worded?

Where to look

Not all guideline development handbooks have a section on identifying existing guidelines or adapting them. This "essential element" might be most obvious in guideline development handbooks meant for international guideline adaptation. Look at the guideline planning

chapters. Examine chapters on equity and additional appendices.
**Illustrative quote**<br>"**Adopting or adapting a guideline**: An 'equity lens' is a series of structured questions focusing on the visibility of equity, inequity and socioeconomic determinants, can be used to assess whether existing guidelines have explicitly considered the needs and values of disadvantaged populations." (*NHMRC*) (28)

Domain 2 – Identifying the guideline group

Incorporate equity considerations into:

- Essential element #4 = Forming guideline development group
- Essential element #5 = Involving special subgroups
- Essential element #6 = Managing conflicts of interest

Essential element #4 Forming guideline development group

Essential element definition: Describes selection of the guideline development group, their roles, and responsibilities. Guideline development committees typically include a chair, a literature review group, voting members, etc. (14)

0<br>Meets none of the criteria NOR considerations	1	2<br>Meets some criteria AND considerations	3	4<br>Meets all criteria AND considerations	999<br>Essential element not in the guideline development handbook

Comments:

How to rate – look for discussion of:
Criteria

1. Involving* special subgroups throughout the guideline process who are either:
    a. Members of the affected special subgroup and representatives OR, if this is not possible (e.g. in instances involving children or people with intellectual disabilities)
    b. Experts on a topic relevant to the subgroups (e.g., experts on inequities in service delivery, epidemiologists, economists, etc.) (28, 29).
2. Defining when special subgroups will participate in guideline development process (e.g. voting, as subcommittee members, etc.) (14)
3. Ensuring that the chair understands, recognizes and mitigates power dynamics within the entire group; the chair encourages participation from entire committee (14, 28, 29)
4. Striving for diverse representation within the guideline development group (e.g. balance of sex, race, expertise etc.) (36, 37) when recruiting panel members.
5. Ensuring the guideline development panel recruitment process is transparent and objective (37-39).

**Involving special subgroups means giving explicit attention by listening to challenges experienced by groups/their representatives during entire guideline development process,*

Additional considerations

1. Are the equity criteria easy to find?
2. Are the equity criteria clearly worded?

Where to look
Look at chapters on planning the guideline. Forming guideline development groups might be titled committee selection or decision-making committee selection. Examine chapters on equity and additional appendices.

Illustrative quote
"Group membership should not only reflect diversity, including geographical, gender balance or cultural background, but should also include populations with where the health conditions are more prevalent…all guidelines groups should try to include at least one indigenous representative…."

(NHMRC, "Determine the Structure and Complexity of the Group,")(28)

Essential element #5 Involving special subgroups

Essential element definition: Engaging group affected by the guideline (e.g. target audience of users). Different terminology might be used: affected subgroups are often referred to as "stakeholders" or "consumers" in many guideline development handbooks.

0<br>Meets none of the criteria NOR considerations	1	2<br>Meets some criteria AND considerations	3	4<br>Meets all criteria AND considerations	999<br>Essential element not in the guideline development handbook

Comments:

How to rate – look for discussion of:
Criteria

1. Recruiting, training, and engaging representatives from relevant subgroups throughout the guideline development process. PROGRESS-PLUS may help stratify these subgroups.
   a. OR if it is not possible (e.g. guidelines involving children), suggestions of alternatives like discussions with other stakeholders (i.e. parents, caregivers, experts).
   b. OR if these alternatives are still not possible, the guideline development handbook suggests systematic review of previous data, etc. (11).
2. Describing structured format for eliciting feedback and participation from special subgroups. For example, structured or semi-structured interviews with special subgroups, systematic reviews of qualitative/quantitative data, etc. (11). An example of a tool to facilitate an interview guide includes the GRADE-FACE series of questions (11, 40)

Additional considerations

1. Are the equity criteria easy to find?
2. Are the equity criteria clearly worded?

Where to look
Information might be in a section on "panel composition." Stakeholders often referred to as "consumers." Examine chapters on equity and additional appendices.

Illustrative quote
"Some approaches to consider include:

- Formal, structured activities (e.g., interviews, focus groups, consensus methods)
- Informal methods (e.g., project or advisory meetings, feedback on documents, public consultations
- Modes of involvement (e.g., face-to-face, online, video-/teleconference, email)
- Timing of involvement (e.g., one-off, multistage, continuous)

You will need to be flexible and adapt to consumer needs for any approach you choose to take, especially if they live remotely or have ongoing health challenges" *(NHMRC, consumer involvement, "Plan for consumer involvement")(28)*

Abbreviation: PROGRESS-PLUS = Place of residence, Race/ethnicity/culture/language, Occupation, Gender/sex, Religion, Education, Socioeconomic Status, Social capital, personal characteristics associated with discrimination (e.g. disability), features of relationships (smoking parents, excluded from school), time dependent relationships.
GRADE-FACE = Grading of Recommendations Assessment, Development and Evaluation–Feasibility, Acceptability, Cost, and Equity Survey

Essential element #6 Managing conflicts of interest

Essential element definition: Defining and managing potential conflicts of interest of guideline development group members.

0<br>Meets none of the criteria NOR considerations	1	2<br>Meets some criteria AND considerations	3	4<br>Meets all criteria AND considerations	999<br>Essential element not in the guideline development handbook

Comments:

How to rate – look for discussion of:

Criteria

1. Identifying financial and non-financial conflict of interest as a source of potential bias that may impact health equity considerations. (11, 41-43). Specific types of interest may include those outlined by Akl et al 2022 (44)
    a. Requiring action related to conflict-of-interest disclosure, for example, specifying when and how a panelist may serve in leadership roles (45).

Additional considerations

1. Is there a policy referenced for managing conflicts of interest (in text or linked)?
2. Is the policy easy to find?
3. Is the policy clearly written?

Where to look

Look for a section labeled conflict of interest. There may be additional information in chapters related to equity. Several organizations have separate policy documents, and the assessor should check the appendix and any additional links.

Illustrative quote

"Can the interest consistently produce bias? Consider whether the interest would produce a consistent direction of bias in the context of guideline development, such as if financial sponsorship were to influence decisions consistently in favor of an intervention or product preferred by the sponsor" (*NMHRC "identifying and managing conflicts of interest")(28)*

Domain 3 – Gathering the evidence

Incorporate equity considerations into:

- Essential element #7 = Establishing clinical questions
- Essential element #8 = Outcomes consideration
- Essential element #9 = Systematic search for evidence and evidence inclusion
- Essential element #10= Summarizing the evidence
- Essential element #11 = Appraising the evidence
- Essential element #12 = Conducing economic evaluation

Essential element #7 Establishing clinical questions

Essential element definition: Identify the key questions that a recommendation should address using a question framework. The most common frameworks are PICO (**P**opulation, **I**ntervention, **C**omparator, and **O**utcomes) and SPICE (**S**etting, **P**erspective, **I**ntervention/exposure/interest, **C**omparison, **E**valuation).

0<br>Meets none of the criteria NOR considerations	1	2<br>Meets some criteria AND considerations	3	4<br>Meets all criteria AND considerations	999<br>Essential element not in the guideline development handbook

Comments:

How to rate – look for discussion of:

Criteria

1. Including questions on both the average health effects of an intervention and the distribution of health effects within a population (11)
2. Considering health equity at each point of the question framework (e.g. PICO or SPICE) (11, 27). This means:
    a. Specifying a population of interest, especially subgroups stratified by any of the PROGRESS PLUS mnemonic
    b. Highlighting interventions aimed at subgroups of the population that reduce the health gradient
    c. Identifying appropriate comparator group(s)
    d. Identifying outcomes that are important to disadvantaged subgroups within the population, either through stakeholder input or expert advice (11)

Additional considerations

1. Are the equity criteria easy to find?
2. Are the equity criteria clearly worded?

Where to look

Look in the sections of guideline development/generating evidence. Establishing clinical questions may follow planning for the guideline and be at the beginning of the guideline development section. Look for additional information on chapters on equity or appendices.

Illustrative quote

For each review question, factors that may affect the outcomes and effectiveness of an intervention, including any wider social factors that may affect health and any health inequalities, should be considered" *(NICE, "Review questions about the effectiveness of an intervention") (29)*

Abbreviation: PICO = Population, Intervention, Comparators, Outcome; SPICE = Setting, Perspective, Intervention, Comparison, Evaluation; PROGRESS-PLUS = Place of residence, Race/ethnicity/culture/language, Occupation, Gender/sex, Religion, Education, Socioeconomic Status, Social capital, personal characteristics associated with discrimination (e.g. disability), features of relationships (smoking parents, excluded from school), time dependent relationships

Essential element #8 Outcomes Consideration

Essential element definition: In the process of guideline development, considering how special subgroups impacted by the recommendation identify possible consequences (20) Special consideration might be given to other social determinants of health.

0<br>Meets none of the criteria NOR considerations	1	2<br>Meets some criteria AND considerations	3	4<br>Meets all criteria AND considerations	999<br>Essential element not in the guideline development handbook

Comments:

How to rate – look for discussion of:

Criteria

1. Involving members of special subgroups/representatives that have been previously identified (same members of special subgroups from essential element 5) when identifying outcomes and potential interventions for the guideline (11). To facilitate broader perspectives on prioritization, this might include involving special subgroups/representatives when rating or ranking the importance of interventions and outcomes or searching databases for evidence on these interventions/outcomes (11).

Additional considerations

1. Are the equity criteria easy to find?
2. Are the equity criteria clearly worded?

Where to look

Outcomes consideration should be defined before systematic review of evidence. Look for additional information in equity chapters or appendices.

Illustrative quote

"**Patient important outcomes** should be explicitly considered along with more narrowly defined clinically important outcomes. It is particularly important to include any potential harm associated with the intervention under review so that a balanced view can be taken at the considered judgement stage." (*SIGN 50, chapter 4, "Defining key questions"*) (46)

Essential element #9 Systematic search for evidence and evidence inclusion

Essential element definition: Creating inclusion and exclusion criteria for evidence, study design, population, interventions, and comparators. Deciding how to identify and obtain evidence.

0<br>Meets none of the criteria NOR considerations	1	2<br>Meets some criteria AND considerations	3	4<br>Meets all criteria AND considerations	999<br>Essential element not in the guideline development handbook

Comments:

How to rate – look for discussion of:

Criteria

1. Searching multiple databases for relevant information (28, 47).
2. Searching non-English studies when appropriate (11)
3. Using search terms with filters/combination of terms related to the subgroup of interest (for instance, include free text/subject headings for geographic locations) when appropriate (11, 25, 47)
4. Including a variety of study types (observational, qualitative, quantitative) when appropriate (11).
5. Drawing from other disciplines when needed to address health concerns of subgroups (11)
6. Acknowledging potential limitations in systematic search for evidence (if found evidence does not have an equity lens), and noting types of missing evidence/missing voices

Additional considerations

1. Are the equity criteria easy to find?
2. Are the equity criteria clearly worded?

Where to look

Look for the evidence generation sections. Look at sections on systematically searching for evidence and/or creating inclusion/exclusion criteria. Also look at sections on how to carry out the systematic search. Examine chapters on equity or other appendices.

Illustrative quote

"Databases relevant to these groups should be identified and searched, such as the *Informit Indigenous Collection* and the *Aboriginal and Torres Strait islander Health Bibliography* (Aboriginal and Torres Strait Islander Health, compiled by the Australian Indigenous Health Infonet), both of which provide searchable databases of literature about Aboriginal and Torres Strait Islander health"
(NHMRC, "Equity," "What to do") (28)

Essential element #10 Summarizing the evidence

Essential element definition: Describes synthesis of the evidence in the form of tables, charts, or brief narratives. May also include additional information that does not fit into all the tables.

0<br>Meets none of the criteria NOR considerations	1	2<br>Meets some criteria AND considerations	3	4<br>Meets all criteria AND considerations	999<br>Essential element not in the guideline development handbook

Comments:

How to rate – look for discussion of:

Criteria

1. Assess differences in baseline risk for an outcome when presenting absolute measures and use appropriate reference groups (11)
2. Summarizing information for relevant subgroups as defined by PROGRESS PLUS when appropriate (25). The guideline development handbook may mention use of tools like PRISMA 2012 equity extension, equity checklist for systematic review authors, and checklist for assessing credibility of subgroup analysis (48-50)
3. When available, presenting both absolute and relative measures of inequality (between relatively advantaged and relatively disadvantaged) (11, 28)
4. Consider GRADE-Equity steps (51). When appropriate, including health equity as an outcome in the summary of finding (SoF) table and including any statements of insufficient evidence (11).

Additional considerations

1. Are the equity criteria easy to find?
2. Are the equity criteria clearly worded?

Where to look

Look for the section following systematic search for evidence. This section might be presented as tables in the guideline development handbook. Examine chapters on health equity or other appendices.

Illustrative quote

"One of the key objectives for evidence synthesis is to explore the reasons for difference in observed effects and to identify any populations or interventions/exposure categories that are associated with these differences. This can be a critical area of investigation used to inform a guideline's recommendations to support specific actions, for different populations. It is especially relevant to considerations of equity" *(NHMRC, "Synthesizing evidence" "Investigate the reasons for different effect")(28)*

Essential element #11 Appraising the evidence

Essential element definition: Assessing the confidence a developer can have by evaluating the "strength and quality" of that evidence. This might include structured approaches like GRADE which assign a "certainty of evidence" by PICO

0<br>Meets none of the criteria NOR considerations	1	2<br>Meets some criteria AND considerations	3	4<br>Meets all criteria AND considerations	999<br>Essential element not in the guideline development handbook

Comments:

How to rate – look for discussion of:

Criteria

1. Assessing indirectness of evidence by considering equity in the GRADE process (11, 15, 51).
2. Acknowledging that certainty of evidence should not be downgraded for indirectness for differences within a population, unless "there are compelling reasons to anticipate differences in effect due to biology/physiology, sociocultural influences, or setting-specific resource issues that impact effectiveness or harms of the intervention" (15)
3. Guidance on assessing confidence for qualitative evidence synthesis using GRADE-CERQual (15, 52)

Additional considerations

1. Are the equity criteria easy to find?
2. Are the equity criteria clearly worded?

Where to look

Look for the section, review of evidence/evidence generation.

Illustrative quote

"The concept of indirectness refers to whether the evidence available, including the population, comparisons and outcomes measured, directly and completely answers the question proposed by the guideline…examples of important indirectness might include:

- Randomized trials of narrow segments of the population such as only participants with relatively mind illnesses or only adult,
- Studies conducted in high-income, urban settings rather than including rural or low-income settings…" *(NHMRC, "Assessing certainty of evidence")(28)*

Abbreviations: GRADE= Grading Recommendations Assessment and Development Evidence; GRADE-CERQUAL- provides a "transparent method for assessing the confidence of evidence from reviews of qualitative research"

Essential element #12 Conducting economic evaluation

Essential element definition: Identifying and analyzing additional economic information. Presenting cost and benefit.

0<br>Meets none of the criteria NOR considerations	1	2<br>Meets some criteria AND considerations	3	4<br>Meets all criteria AND considerations	999<br>Essential element not in the guideline development handbook

Comments:

How to rate – look for discussion of:

Criteria

1. Including methods to incorporate equity into guideline development during economic analysis phase. These methods might include equity weighting, extended or distributional cost-effectiveness analysis, economic analysis by subgroups, etc. (29).
2. Economic evaluations should be carried out for each of the relevant subgroups identified by PROGRESS-PLUS OR developers provide justification of why these analyses were not performed

Additional considerations

1. Is the section on equity considerations of economic evaluation easy to find?
2. Is the section on equity in economic evaluations clearly worded?

Where to look:
Not all documents will have a section on conducting economic analysis, since this is prioritized in countries where the government is the health payer. Look for sections marked economic analysis. Examine chapters on equity and additional appendices.

Illustrative quote
"To support our commitment to addressing health inequalities, we have commissioned a prototype tool to explore the approach of providing quantitative estimates of the impact of NICE recommendations on health inequalities. The tool uses distributional cost-effectiveness analysis to model changes in health inequalities between 5 socioeconomic groups in England based on the neighborhood index of multiple deprivation" *(NICE, "Equity considerations" pg. 188) (29)*

Abbreviations: PROGRESS-PLUS= [1]PROGRESS-PLUS = Place of residence, Race/ethnicity/culture/language, Occupation, Gender/sex, Religion, Education, Socioeconomic Status, Social capital, personal characteristics associated with discrimination (e.g. disability), features of relationships (smoking parents, excluded from school), time dependent relationships

Domain 4 – Drafting and review

Incorporate equity considerations into:

- Essential element #13= Creating recommendations
- Essential element #14= Wording and considering ethics
- Essential element #15= Reporting and consulting experts

Essential element #13 Creating recommendations

Essential element definition: Includes interpreting evidence and developing recommendations using a structured approach (like the Evidence to Decision framework). Strength of the recommendations refers to a judgement about confidence that the recommendation will benefit people.

0<br>Meets none of the criteria NOR considerations	1	2<br>Meets some criteria AND considerations	3	4<br>Meets all criteria AND considerations	999<br>Essential element not in the guideline development handbook

Comments:

How to rate – look for discussion of:

Criteria

1. Balancing benefits and harms (i.e. identifying potential unintended consequences like widening inequities) of proposed interventions to special subgroups (11, 25, 29) (Note: this requires some judgement from panel members when evidence is lacking or poor quality).
2. Creating equity-focused recommendations through consideration of interventions and their impacts on special subgroups (11)
    a. Consulting special subgroup individuals or representatives when creating recommendations
3. Consider using one of the equity-focused recommendations frameworks when relevant (for instance WHO-INTEGRATE EtD framework or application of GRADE equity questions to the EtD framework) (11, 51, 53)

Additional considerations

1. Are the equity criteria easy to find?
2. Are the equity criteria clearly worded?

Where to look

Look for the section, review of evidence/evidence generation.

Illustrative quote

The effect of an intervention on equity is one of the factors that determine the strength of a recommendation: if an intervention is likely to reduce health inequities, a strong recommendation may be warranted, provided it is justifiable in light of other factors. On the other hand, if equity is a key consideration and evidence on the intervention's potential effects on equity is sparse, a conditional recommendation may be appropriate. In the latter case particularly, the guideline should outline the key gaps in knowledge and provide an agenda for future research. Evaluation and monitoring of the impact of recommendations that potentially affect inequities are also critically important and should be

articulated in the guideline document." (*WHO, Chapter 5, "Developing recommendations") (27)*

Abbreviations: WHO-INTEGRATE has 6 criteria: balance of health benefits and harms, human rights and sociocultural acceptability, health equity, equality and nondiscrimination, societal implications, financial and economic considerations, and feasibility and health system considerations

Essential element #14 Wording and considering ethics

Essential element definition: Has to do with the wording and syntax of recommendations as it relates to ethics including equity considerations.

0<br>Meets none of the criteria NOR considerations	1	2<br>Meets some criteria AND considerations	3	4<br>Meets all criteria AND considerations	999<br>Essential element not in guideline development handbook

Comments:

How to rate – look for discussion of:

Criteria

1. Avoiding language that may cause harm for certain population subgroups (11, 25, 54)
2. Using inclusive language when defining special subgroups. This involves highlighting social determinants of health over simplifying terms based on race, gender, etc. (11, 55). For example, specifying gender by delineating cisgender and others.
3. Translating the guideline into local language when relevant (27).

Additional considerations

1. Are the equity criteria easy to find?
2. Are the equity criteria clearly worded?

Where to look

Look for the section on wording of guidelines. Guideline development handbooks do not often focus on dictating wording outside of recommendation development. Information on wording may be in in a policy that the organization refers to elsewhere on their website or in appendices. Wording might also be included throughout the handbook.

Illustrative quote

"Throughout the process careful attention should be paid to the use of language, the selection of case examples, the expressions used to refer to population groups, the choice of photographs, etc. Setting the ground rules up front is much easier than trying to modify a document that is ready for layout. In translations of guidelines, gender-sensitive and non-stigmatizing language should always be employed." (*WHO, chapter 5 "producing and publishing the guideline, section 5.4.3.1) (27)*

Essential element #15 Reporting and consulting experts

Essential element definition: Refers to how a draft recommendation will be assessed before implementation by stakeholders and members of the guideline review committee

0<br>Meets none of the criteria NOR considerations	1	2<br>Meets some criteria AND considerations	3	4<br>Meets all criteria AND considerations	999<br>Essential element not in guideline development handbook

Comments:

How to rate – look for discussion of:

Criteria

1. Sharing final recommendations with special groups including consultation with representative organizations and individuals, and invite these groups for comment
    a. Commenting might be through 1) public meeting, 2) submission for public consultation or 3) posted on website for comment, (24, 25, 28, 29)
2. Incorporating suggestions from special subgroups and/or representatives of individuals with lived experience into final version of recommendations. These suggestions might be related to acceptability and feasibility from an equity perspective from subgroups that are systematically and structurally disadvantaged.

Additional considerations

1. Are the equity criteria easy to find?
2. Are the equity criteria clearly worded?

Where to look

Reporting and consulting experts should come before guideline evaluation and updating. Examine chapters on health equity and any additional appendices.

Illustrative quote

"Public consultation methods can be either open, targeted or a combination of the two (GIN Public 2012). By inviting specific stakeholders to comment, targeted consultation methods enable feedback to be sought in a relatively controlled manner; however, this also runs the risk of important viewpoints being overlooked. Open consultation is more transparent and can capture views from individuals or groups that might not otherwise have been planning or expected to engage." (*NHMRC*) (28)

Domain 5 – Dissemination, implementation, evaluation, and updating

Incorporate equity considerations into:

- Essential element #16= Dissemination and publications
- Essential element #17= Implementation and evaluation
- Essential element #18= Updating

Essential element #16 Dissemination and publications

Essential element definition: Describes publication of guideline in different formats to ensure "relevant groups" are aware of their publication

0<br>Meets none of the criteria NOR considerations	1	2<br>Meets some criteria AND considerations	3	4<br>Meets all criteria AND considerations	999<br>Essential element not in guideline development handbook

Comments:

How to rate – look for discussion of:

Criteria

1. Having clear dissemination products depending on the target audience. This might include multiple publication formats and translations of a guideline or recommendations (including those that are directed to specific types of health professionals or to specific patient or special subgroups) to ensure a wide outreach
   a. Potential formats might include peer review publication, short guideline summary, technical report, lay versions (like patient pages), (56), online or in print version. The guideline development handbook may specify that these alternative formats be created by guideline developers themselves to avoid losing intent and meaning of the recommendations.

Additional considerations

1. Are the equity criteria easy to find?
2. Are the equity criteria clearly worded?

Where to look

Look at the sections on implementation, evaluation, and updating. This information may not have its own section but be part of other sections like updating or adapting guidelines. Examine sections on equity and additional annexes.

Illustrative quote

"Patients as champions for change

Patients are a powerful agent for change in the health service. Guidelines are published with an accompanying patient and carer version of the guideline and by being aware of a clinical guideline, patients can ask for their care to be in line with the latest recommendations. Making use of connections with patient groups and voluntary organisations also affords more opportunities to raise awareness of guidelines. Lay representatives on guideline development groups are supported to raise awareness at conferences and other events." *(SIGN50, chapter 10, "Implementation")* (46)

Essential element #17 Implementation and evaluation

Essential element definition: Describes how guidelines are put into practice, including strategies so that audience of the guideline are aware to enhance uptake

0<br>Meets none of the criteria NOR considerations	1	2<br>Meets some criteria AND considerations	3	4<br>Meets all criteria AND considerations	999<br>Essential element not in guideline development handbook

Comments:

How to rate – look for discussion of:

Criteria

1. Creating tools/systems that monitor guideline recommendations uptake in the audience that serves special subgroups (11)
    a. This might include training and decision support tools on the new guideline with health professional groups that have responsibility of implementing these guidelines
2. Using indicators stratified by population subgroup characteristics to monitor health disparities or measure implementation within subgroup (11). This could mean collecting surveillance data on relevant health outcomes and indicators and consulting special interest groups for population subgroups to obtain feedback (11).

Additional considerations

1. Are the equity criteria easy to find?
2. Are the equity criteria clearly worded?

Where to look

Look for the section, review of evidence/evidence generation.

Illustrative quote

"Topic experts (including members of NICE's Expert Advisers Panel) are invited to participate in surveillance. They provide their views about how an event affects the recommendations, and their knowledge of recent developments in the topic area. If the response from topic experts is limited, or further specialist input is needed, we may seek input from other experts." *(NICE, chapter 13, "Surveillance and assessment process)(29)*

Essential element #18 Updating

Essential element definition: Describes how and when a guideline needs revision based on availability of new evidence or other factors(20)

0<br>Meets none of the criteria NOR considerations	1	2<br>Meets some criteria AND considerations	3	4<br>Meets all criteria AND considerations	999<br>Essential element not in guideline development handbook

Comments:

How to rate – look for discussion of:

Criteria

1. Monitoring new evidence on changes to epidemiology and intervention that may impact subgroups of the population and present this evidence and include it in the update. Practically, an update might mean new systematic review, consideration of the EtD process, or judgment on old recommendations all with an equity lens.
    a. Encourage collection of new evidence on potential benefits, harms and acceptability of, and access or utilization barriers to currently recommended interventions for a subgroup; especially if data are sparse
2. Including special subgroups in the entire updating process (either for an entire guideline or partial update). The updating process includes: forming groups to update guideline, identifying new evidence, assessment for the need to update, updating process, external review, and publication (57, 58)

Additional considerations

1. Are the equity criteria easy to find?
2. Are the equity criteria clearly worded?

Where to look

Look for sections on implementation, evaluation, and updating. Examine chapters on equity and any additional appendices.

Illustrative quote

"In cases where new and potentially relevant evidence has been identified, the co-chairs need to assess whether this new evidence warrants a modification of current recommendations. Situations in which an evidence-based guideline necessitates updating include changes in available interventions, in evidence on benefits or harms of available interventions, in new population targeted, in the values placed on important outcomes, in the resources available in healthcare, or if the evidence results in practice changes." *(IDSA, chapter 16, "Updating and retiring guidelines")(59)*

References

1. Steinberg E, Greenfield S, Wolman DM, Mancher M, Graham R. Clinical practice guidelines we can trust: National Academies Press; 2011.
2. Turner T, Misso M, Harris C, Green S. Development of evidence-based clinical practice guidelines (CPGs): comparing approaches. Implement Sci. 2008 Oct 27;3(1):45.
3. Rosenfeld RM, Shiffman RN, Robertson P. Clinical Practice Guideline Development Manual, Third Edition: A Quality-Driven Approach for Translating Evidence into Action. Otolaryngology-Head and Neck Surgery. 2013 Jan;148(1_suppl):S1-S55.
4. Whitehead M. The concepts and principles of equity and health. Health promotion international. 1991;6(3):217-28.
5. Braveman P, Gruskin S. Defining equity in health. Journal of Epidemiology & Community Health. 2003;57(4):254-8.
6. Sen A, Anand S, Peter F. Why health equity? : Oxford University Press; 2004. p. 21-33.
7. Anand S. The concern for equity in health. Journal of Epidemiology and Community Health. 2002;56(7):485.
8. Persaud N. Clinical practice guideline recommendations can promote or undermine health equity. Clinical and Public Health Guidelines. 2025;2(2):e70011.
9. Mizen LA, Macfie ML, Findlay L, Cooper SA, Melville CA. Clinical guidelines contribute to the health inequities experienced by individuals with intellectual disabilities. Implement Sci. 2012 May 11;7(1):42.
10. Chan V, Estrella MJ, Hanafy S, Colclough Z, Joyce JM, Babineau J, et al. Equity considerations in clinical practice guidelines for traumatic brain injury and homelessness: a systematic review. EClinicalMedicine. 2023 Sep;63:102152.
11. Shaver N, Bennett A, Beck A, Skidmore B, Traversy G, Brouwers M, et al. Health equity considerations in guideline development: a rapid scoping review. CMAJ Open. 2023 Mar-Apr;11(2):E357-E71.
12. Dans AM, Dans L, Oxman AD, Robinson V, Acuin J, Tugwell P, et al. Assessing equity in clinical practice guidelines. J Clin Epidemiol. 2007 Jun;60(6):540-6.
13. Pottie K, Magwood O, Rahman P, Concannon T, Alonso-Coello P, Garcia AJ, et al. GRADE Concept Paper 1: Validating the "FACE" instrument using stakeholder perceptions of feasibility, acceptability, cost, and equity in guideline implement. Journal of Clinical Epidemiology. 2021 Mar;131:133-40.
14. Akl EA, Welch V, Pottie K, Eslava-Schmalbach J, Darzi A, Sola I, et al. GRADE equity guidelines 2: considering health equity in GRADE guideline development: equity extension of the guideline development checklist. J Clin Epidemiol. 2017 Oct;90:68-75.
15. Welch VA, Akl EA, Pottie K, Ansari MT, Briel M, Christensen R, et al. GRADE equity guidelines 3: considering health equity in GRADE guideline development: rating the certainty of synthesized evidence. J Clin Epidemiol. 2017 Oct;90:76-83.
16. Pottie K, Welch V, Morton R, Akl EA, Eslava-Schmalbach JH, Katikireddi V, et al. GRADE equity guidelines 4: considering health equity in GRADE guideline development: evidence to decision process. J Clin Epidemiol. 2017 Oct;90:84-91.
17. Lin JS, Webber EM, Bean SI, Evans CV. Development of a Health Equity Framework for the US Preventive Services Task Force. JAMA Netw Open. 2024 Mar 4;7(3):e241875.
18. Dewidar O, Sayfi S, Pardo JP, Welch V, Wright GC, Akl EA, et al. Enhancing health equity considerations in guidelines: health equity extension of the GIN-McMaster Guideline Development Checklist. EClinicalMedicine. 2025 Apr;82:103135.
19. Ansari S, Rashidian A. Guidelines for guidelines: are they up to the task? A comparative assessment of clinical practice guideline development handbooks. PLoS One. 2012;7(11):e49864.

20. Schunemann HJ, Wiercioch W, Etxeandia I, Falavigna M, Santesso N, Mustafa R, et al. Guidelines 2.0: systematic development of a comprehensive checklist for a successful guideline enterprise. CMAJ. 2014 Feb 18;186(3):E123-42.
21. Brouwers MC, Kho ME, Browman GP, Burgers JS, Cluzeau F, Feder G, et al. AGREE II: advancing guideline development, reporting and evaluation in health care. CMAJ. 2010 Dec 14;182(18):E839-42.
22. AGREE Next Steps Consortium (2017). The AGREE II Instrument [Electronic version]. [cited 2025 July 22]; Available from: http://www.agreetrust.org
23. Brouwers MC, Kho ME, Browman GP, Burgers JS, Cluzeau F, Feder G, et al. AGREE II: advancing guideline development, reporting and evaluation in health care. Cmaj. 2010;182(18):E839-E42.
24. Scottish Intercollegiate Guidelines Network (SIGN) A guideline developer's handbook Edinburgh: SIGN; 2019. (SIGN publication no. 50). [November 2019]. Available from: http://www.sign.ac.uk.
25. Lin JS, Webber EM, Bean SI, Evans CV. Development of a Health Equity Framework for the US Preventive Services Task Force. JAMA Network Open. 2024;7(3):e241875-e.
26. Oliver S, Kavanagh J, Caird J, Lorenc T, Oliver K, Harden A, et al. Health promotion, inequalities and young people's health: a systematic review of research 2008 Available from: https://researchonline.lshtm.ac.uk/id/eprint/2603/1/Health%20promotion,%20inequalities%20and%20young%20people's%20health%20a%20systematic%20review%20of%20research.pdf.
27. The World Health Organization [Internet] WHO Handbook for Guidelines Development, 2nd ed. World Health Organization 2014 [cited 2025 July 4] Available from: https://iris.who.int/handle/10665/145714.
28. National Health and Medical Research Council [Internet] Guidelines for guidelines handbook [cited 2025 Nov 26]. Available from: https://www.nhmrc.gov.au/guidelinesforguidelines.
29. National Institute for Health and Care Excellence [Internet] Developing NICE guidelines: the manual [cited 2025 Nov 26]. Available from: https://www.nice.org.uk/process/pmg20/chapter/introduction.
30. Dans AM, Dans L, Oxman AD, Robinson V, Acuin J, Tugwell P, et al. Assessing equity in clinical practice guidelines. Journal of clinical epidemiology. 2007;60(6):540-6.
31. Maaløe N, Ørtved AMR, Sørensen JB, Dmello BS, van den Akker T, Kujabi ML, et al. The injustice of unfit clinical practice guidelines in low-resource realities. The Lancet Global Health. 2021;9(6):e875-e9.
32. Maaløe N, Housseine N, Sørensen JB, Obel J, Sequeira DMello B, Kujabi ML, et al. Scaling up context-tailored clinical guidelines and training to improve childbirth care in urban, low-resource maternity units in Tanzania: A protocol for a stepped-wedged cluster randomized trial with embedded qualitative and economic analyses (The PartoMa Scale-Up Study). Global health action. 2022;15(1):2034135.
33. Craig P, Di Ruggiero E, Frolich KL, Mykhalovskiy E, White M, Campbell R, et al. Taking account of context in population health intervention research: guidance for producers, users and funders of research. Canadian Institutes of Health Research (CIHR)–National Institute for Health Research (NIHR) Context Guidance Authors Group Southampton: National Institute for Health Research https://www.ncbi.nlm.nih.gov/books/NBK498645/
34. McCaul M, Ernstzen D, Temmingh H, Draper B, Galloway M, Kredo T. Clinical practice guideline adaptation methods in resource-constrained settings: four case studies from South Africa. BMJ Evidence-Based Medicine. 2020;25(6):193-8.
35. Sagam CK, Were L, Otieno JA, Mulaku M, Kariuki S, Ochodo EA. Quality assessment of clinical practice guidelines in Kenya using the AGREE II tool: methodological review protocol. 2022.
36. Kamran SC, Pompa IR, Niemierko A, Dawes SL, Zaky SS, Deville Jr C. Demographic Trends Among American Society for Radiation Oncology Clinical Practice Guideline Task Force Participants From 2010 to 2022. International Journal of Radiation Oncology* Biology* Physics. 2023;116(2):257-69.

37. Persaud N, Ally M, Woods H, Workentin A, Baxter NN, Boozary A, et al. Racialised people in clinical guideline panels. The Lancet. 2022;399(10320):139-40.
38. Bohren MA, Javadi D, Vogel JP. Gender balance in WHO panels for guidelines published from 2008 to 2018. Bulletin of the World Health Organization. 2019;97(7):477.
39. Merman E, Pincus D, Bell C, Goldberg N, Luca S, Jakab M, et al. Differences in clinical practice guideline authorship by gender. Lancet (London, England). 2018;392(10158):1626-8.
40. Pottie K, Magwood O, Rahman P, Concannon T, Alonso-Coello P, Garcia AJ, et al. GRADE concept paper 1: validating the "FACE" instrument using stakeholder perceptions of feasibility, acceptability, cost, and equity in guideline implement. Journal of Clinical Epidemiology. 2021;131:133-40.
41. Bindslev JBB, Schroll J, Gøtzsche PC, Lundh A. Underreporting of conflicts of interest in clinical practice guidelines: cross sectional study. BMC medical ethics. 2013;14(1):1-7.
42. Norris SL, Holmer HK, Ogden LA, Selph SS, Fu R. Conflict of interest disclosures for clinical practice guidelines in the national guideline clearinghouse. PloS one. 2012;7(11):e47343.
43. National Institute for Health and Care Excellence [Internet] Policy on declaring and managing interests for NICE advisory committees: also inclues witnesses, expert commentators and other contributors v1.6 c2018 [cited 2025 Nov 26]. Available from: https://www.nice.org.uk/Media/Default/About/Who-we-are/Policies-and-procedures/declaration-of-interests-policy.pdf.
44. Akl EA, Hakoum M, Khamis A, Khabsa J, Vassar M, Guyatt G. A framework is proposed for defining, categorizing, and assessing conflicts of interest in health research. Journal of clinical epidemiology. 2022;149:236-43.
45. Ngo-Metzger Q, Moyer V, Grossman D, Ebell M, Woo M, Miller T, et al. Conflicts of interest in clinical guidelines: update of US Preventive Services Task Force policies and procedures. American journal of preventive medicine. 2018;54(1):S70-S80.
46. Scottish Intercollegiate Guidelines Network (SIGN). A guideline developer's handbook. Edinburgh: SIGN; 2019. : (SIGN publication no. 50). [November 2019]. .
47. Welch VA, Petkovic J, Jull J, Hartling L, Klassen T, Kristjansson E, et al. Chapter 16: Equity and specific populations. Cochrane Handbook for Systematic Reviews of Interventions version. 2019;6(2).
48. Welch V, Petticrew M, Tugwell P, Moher D, O'Neill J, Waters E, et al. PRISMA-Equity 2012 extension: reporting guidelines for systematic reviews with a focus on health equity. PLoS medicine. 2012;9(10):e1001333.
49. Ueffing E TP, Welch V, Petticrew M, Kristjansson E for the Campbell and Cochrane Equity Methods Group. [Internet] Equity Checklist for Systematic Review Authors. Version 2012-10-02. [2025 Jan 4]. Available from: https://methods.cochrane.org/sites/methods.cochrane.org.equity/files/uploads/EquityChecklist2012.pdf.
50. Sun X, Briel M, Walter SD, Guyatt GH. Is a subgroup effect believable? Updating criteria to evaluate the credibility of subgroup analyses. Bmj. 2010;340.
51. Dewidar O, Pardo JP, Welch V, Hazlewood GS, Darzi AJ, Barnabe C, et al. Operationalizing the GRADE-equity criterion to inform guideline recommendations: application to a medical cannabis guideline. Journal of Clinical Epidemiology. 2024;165:111185.
52. Wainwright M, Zahroh RI, Tunçalp Ö, Booth A, Bohren MA, Noyes J, et al. The use of GRADE-CERQual in qualitative evidence synthesis: an evaluation of fidelity and reporting. Health Research Policy and Systems. 2023;21(1):77.
53. Rehfuess EA, Stratil JM, Scheel IB, Portela A, Norris SL, Baltussen R. The WHO-INTEGRATE evidence to decision framework version 1.0: integrating WHO norms and values and a complexity perspective. BMJ Global Health. 2019;4(Suppl 1):e000844.
54. Association AM. Advancing health equity: A guide to language, narrative and concepts. AMA website. 2021:685-91.
55. Siddique SM, May FP. Race-based clinical recommendations in gastroenterology. Gastroenterology. 2022;162(2):408-14. e2.

56. Shekelle P, Woolf S, Grimshaw JM, Schünemann HJ, Eccles MP. Developing clinical practice guidelines: reviewing, reporting, and publishing guidelines; updating guidelines; and the emerging issues of enhancing guideline implementability and accounting for comorbid conditions in guideline development. Implementation Science. 2012;7(1):1-7.
57. Petkovic J, Riddle A, Lytvyn L, Khabsa J, Akl EA, Welch V, et al. PROTOCOL: Guidance for stakeholder engagement in guideline development: A scoping review. Campbell Systematic Reviews. 2022;18(2):e1242.
58. Vernooij RW, Sanabria AJ, Sola I, Alonso-Coello P, Martinez Garcia L. Guidance for updating clinical practice guidelines: a systematic review of methodological handbooks. Implement Sci. 2014 Jan 2;9(1):3.
59. Infectious Disease Society of America [Internet] Handbook on Clinical Practice Guideline Development [cited 2025 Nov 26]. Available from: https://www.idsociety.org/globalassets/idsa/topics-of-interest/lyme/idsa-handbook-on-cpg-development-10.15.pdf.
